# Elements and COVID-19: A Comprehensive Overview of Studies on Their Blood/Urinary Levels and Supplementation with an Update on Clinical Trials

**DOI:** 10.3390/biology11020215

**Published:** 2022-01-28

**Authors:** Agnieszka Ścibior, Ewa Wnuk

**Affiliations:** Laboratory of Oxidative Stress, Centre for Interdisciplinary Research, The John Paul II Catholic University of Lublin, 20-708 Lublin, Poland; ewa.wnuk@kul.pl

**Keywords:** COVID-19, biomarkers, clinical features, hematological/biochemical alterations, therapeutic strategies, elements, clinical trials

## Abstract

**Simple Summary:**

COVID-19 is a disease caused by the SARS-CoV-2 coronavirus spreading mainly through person-to-person contact. It has caused millions of deaths around the world and lasting health problems in individuals who have survived the disease. This review concisely summarizes certain issues related to COVID-19 with a focus on elements and gives an update on clinical trials where some minerals will be tested/have been tested alone or in combination with drugs, vitamins, or plant extracts/herbal formulations in COVID-19 patients and in those at higher COVID-19 risk.

**Abstract:**

The current report provides a brief overview of the clinical features, hematological/biochemical abnormalities, biomarkers, and AI-related strategies in COVID-19; presents in a nutshell the pharmacological and non-pharmacological therapeutic options; and concisely summarizes the most important aspects related to sociodemographic and behavioral factors as well as comorbidities having an impact on this disease. It also gives a brief outline of the effect of selected elements on immune response and collects data on the levels of micro-/macro-elements and toxic metals in the blood/urine of SARS-CoV-2 infected patients and on supplementation with minerals in COVID-19 subjects. Moreover, this review provides an overview of clinical trials based on the use of minerals alone or in combination with other agents that can provide effective responses toward SARS-CoV-2 infection. The knowledge compiled in this report lays the groundwork for new therapeutic treatments and further research on biomarkers that should be as informative as possible about the patient’s condition and can provide more reliable information on COVID-19 course and prognosis. The collected results point to the need for clarification of the importance of mineral supplementation in COVID-19 and the relationships of the levels of some minerals with clinical improvement.

## 1. Introduction

In December 2019, a novel coronavirus disease—i.e., COVID-19—caused by a betacoronavirus (β-CoV) of unknown origin called Severe Acute Respiratory Syndrome Coronavirus 2 (SARS-CoV-2) broke out in Hubei province of China (Wuhan) and rapidly spread worldwide, leading to a global pandemic. The dispersion of the single-stranded positive-sense RNA virus via the respiratory tract made human-to-human aerosol transmission the major source of the infection [1,2]. At present (i.e., 18 January 2022), COVID-19 is affecting 220 countries with over 326 million confirmed cases and more than 5.5 million deaths [3].

**Figure 1:**
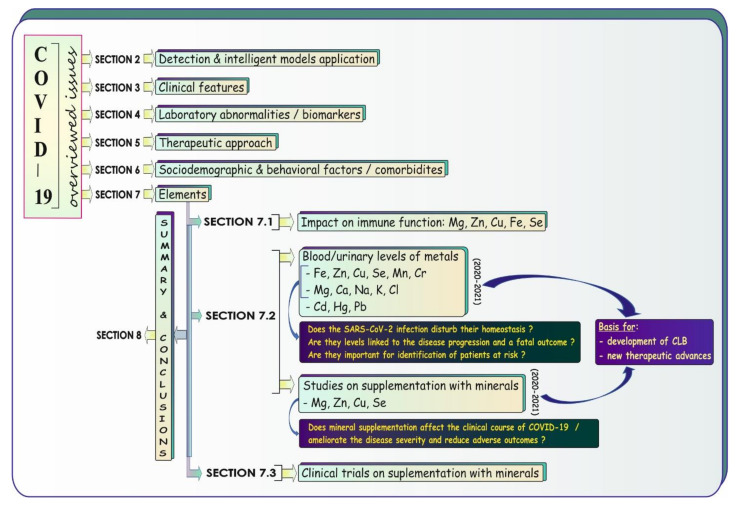
Graphical summary of the overviewed COVID-19 issues. CLB: clinical laboratory biomarkers.

As COVID-19 has an impact on everyone’s life, in the present review we tried to summarize certain issues related to the SARS-CoV-2 pandemic, focusing on elements in/for COVID-19, in a form accessible to anyone interested in COVID-19 in general. After the Introduction, the article consists of eight main sections presented in Figure 1. We aimed to provide a brief overview of the available diagnostic tools for detection/monitoring of SARS-CoV-2 infection and artificial intelligence (AI)-related models helpful in COVID-19 management (Section 2), clinical features of COVID-19 (Section 3), laboratory abnormalities and biomarkers crucial in the monitoring of COVID-19 patients (Section 4), therapeutic strategies for this illness (Section 5), some sociodemographic/behavioral factors and certain comorbidities affecting COVID-19 (Section 6), and some issues related to elements in COVID-19 (Section 7 with Section 7.1, Section 7.2 and Section 7.3). Section 7.1 provides a concise summary of the immunomodulatory potential of selected minerals, Section 7.2 collects data obtained from studies on the blood/urinary levels of some metals in COVID-19 patients and findings of research on supplementation of COVID-19 subjects with micronutrients, and the last part—i.e., Section 7.3—overviews the registered clinical trials undertaken with the aim to investigate the effects of administration of minerals alone or in combination with other agents in COVID-19 patients and COVID-19 risk groups. Finally, Section 8 consists of a summary and conclusions.

One of the main goals of this review is to provide concise knowledge of the levels of some elements in positive COVID-19 patients. Hence, we have overviewed the most important literature presenting findings of recent research focused on examination of the concentration of metals in the blood and urine of mild, moderate, severe, and critically ill COVID-19 subjects and their relationships with the COVID-19 course. In the analysis of the collected data, we aimed to obtain a clear answer to the following questions: (a) Does SARS-CoV-2 infection disturb the homeostasis of some elements?; (b) Are their levels linked to the disease progression and a fatal outcome?; and (c) Are they important for identification of patients at risk? Additionally, we have sought an unequivocal answer to the question whether (d) mineral supplementation affects the clinical course of COVID-19/ameliorates the disease severity and reduces adverse outcomes. As some elements have an impact on immune response, it can be assumed that they may influence the response to SARS-CoV-2 infection. Hence, we have reviewed original articles on the effects of supplementation with essential elements in COVID-19 patients. The current scenario for clinical (interventional) studies, which can provide effective responses to COVID-19, was overviewed as well. Clinical trials with minerals which will be tested/have been tested alone or in combination with other agents in COVID-19 patients and those at higher COVID-19 risk were of our special interest.

### Methodology

Web of Science and PubMed were mainly used to collect articles related to the topic. The keywords used to obtain data on the levels of some metals in COVID-19 patients and on supplementation of COVID-19 subjects with some minerals included ‘trace element*COVID-19’, ‘micronutrient*COVID-19’, ‘electrolyte imbalance*COVID-19’, ‘zinc*COVID-19’, ‘iron*COVID-19’, ‘metals*COVID-19 severity’, ‘clinical characteristic*COVID-19’, ‘clinical features*COVID-19’, ‘COVID-19*zinc supplementation’, ‘COVID-19*copper supplementation’, ‘COVID-19*selenium supplementation’, ‘COVID-19*magnesium supplementation’, ‘COVID-19*calcium supplementation’. Only original journal articles (published in 2020–2021) presenting human studies written in English were reviewed.

In turn, the website maintained by the National Library of Medicine (NLM) at the National Institutes of Health (NIH) (https://clinicaltrials.gov/; accessed on 14 January 2022) [4] and Cochrane’s COVID-19 Study Register (https://COVID-19.cochrane.org/; accessed on 14 January 2022) [5] were used to find registered clinical trials (including those not yet recruiting/active not recruiting, recruiting, and completed) on the administration of minerals (alone or in combination with drugs, vitamins, or plant extracts/herbal formulations containing natural antioxidants) to COVID-19 subjects or individuals at higher COVID-19 risk. The following keywords were used ‘COVID-19*zinc’, ‘COVID-19*copper’, ‘COVID-19*magnesium’, and ‘COVID-19*selenium’. Selected information about minerals (i.e., the type of compound, dose, frequency, and duration of administration) and participants (number, age, sex) included in clinical trials along with a unique identification code in the NCT, CTRI, ACTRN, PACRT, and IRCT format are presented in a tabular form. Additionally, we used certain websites (i.e., www.who.int; www.ema.europa.eu; www.fda.gov; accessed on 14 January 2022) [6,7,8] to collect data on some issues related to the topic of interest.

## 2. COVID-19: Detection and intelligent Models Application—A Brief Note

It is known that early detection of SARS-CoV-2 and prompt treatment are important to control the spread of the virus and prevent the epidemic. Currently, the diagnostic strategy used to confirm the COVID-19 infection includes a molecular assay, i.e., the reverse-transcription polymerase chain reaction (RT-PCR), which is the preferred method for testing [9]. As an alternative, a CRISPR-Cas 12-based assay has been developed to detect SARS-CoV-2 from respiratory swab RNA extracts [10]. A well-known common biochemical technique, i.e., an enzyme-linked immunosorbent assay (ELISA) detecting antibody responses specific to SARS-CoV-2 [11], is another diagnostic tool. Additionally, chest X-ray (CXR) which plays an important role in the diagnosis of COVID-19 and computed tomography (CT) which useful for monitoring disease progression are also often used, although the application of these diagnostic imaging techniques has certain recommendations [9], the description of which are omitted in this report. Moreover, lung ultrasonography (US), which plays a crucial role for rapid assessment of the severity of SARS-CoV-2 pneumonia/acute respiratory distress syndrome (ARDS) [12], is employed as well [13].

It should also be highlighted that, due to its numerous advantages, the application of artificial intelligence (AI), which is a rapidly developing internal branch of computer science, is becoming increasingly popular in the management of COVID-19. AI is a valuable tool for analysis of data related to this illness and can be helpful when a rapid diagnosis and a decision to undertake appropriate treatment are required. As part of AI-related strategies, some deep learning (DL) methods (consisting of numerous of algorithms)—including generative adversarial networks (GANs), extreme learning machine (ELM), and long-short term memory (LSTM)—have been developed to be used in detection and diagnosis of COVID-19. They can help monitor patients and predict effective strategies of treatment (e.g., in a high-risk group of patients with certain comorbidities). They can also estimate the treatment effectiveness and probability of stay in hospital or intensive care unit (ICU) and allow detecting coronavirus when traditional approaches have been ineffective [14]. Moreover, some models—e.g., a novel variational-LSTM autoencoder model introduced by Ibrahim et al. [15] based on DL—can be used to forecast the spread of COVID-19 at the global and country levels. Last but not least, AI techniques can also improve the drug or vaccine development process, which could be faster, cheaper, and more effective [16,17].

## 3. COVID-19: Clinical Characteristics—A Brief Summary

**Figure 2:**
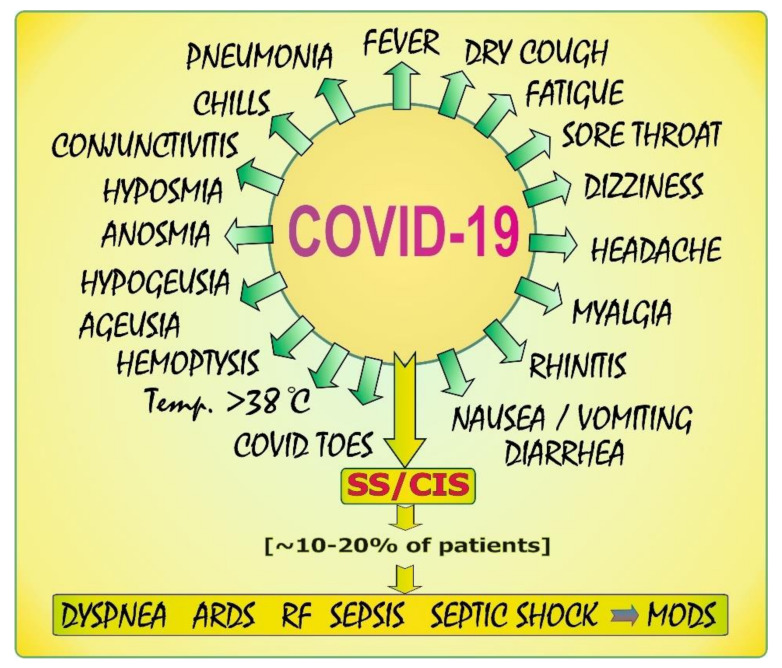
COVID-19: symptoms. Elaborated on the basis of available literature data [19,20,21,22]. ARDS: acute respiratory distress syndrome; RF: respiratory failure; SS: severe symptoms; CIS: critical illness symptoms; MODS: multiple organ dysfunction syndrome.

As reported by Centers for Disease Control and Prevention [18], there is a wide range of COVID-19 symptoms that may appear within 2–14 days, most often 5–6 days [6] after exposure to the virus. In Figure 2, clinical manifestations linked to COVID-19 are concisely summarized.

According to the current WHO data [6], the most common symptoms of COVID-19 include fever, dry cough, and fatigue. In patients with mild infection, especially in children and adolescents, COVID toes were commonly observed [23]. Other symptoms such as chills, dizziness, headache, sore throat, conjunctivitis, myalgia, a diminished sense or loss of taste (i.e., hypogeusia and ageusia, respectively), a decreased ability to detect odors (hyposmia) or total loss of olfactory function (anosmia), and gastrointestinal disturbances such as diarrhea, nausea, and vomiting were reported to be the less common clinical manifestations [6]. Rhinitis and hemoptysis were classified as rare symptoms [22]. Approximately 10–20% of patients are characterized by the presence of severe/critical illness symptoms (SS/CIS) such as dyspnea and sepsis-related ARDS which, in extreme cases, may result in multiple organ dysfunction syndrome (MODS) [19,24,25].

Certain literature reports draw attention to the relationship between some symptoms and the COVID-19 course. For example, Cao et al. [26] noted that a temperature over 38.5 °C is related to the severity of the illness. Gao et al. [27], who reviewed the risk factors for severe and critically ill COVID-19 patients, referred to data on the higher risk of mechanical ventilation in patients with a temperature above 38 °C. According to the information provided by WHO [6], a high temperature (i.e., >38 °C) is indicative of the severe symptoms of COVID-19 disease. It should be emphasized that the clinical manifestations vary from asymptomatic to acute respiratory distress depending, e.g., on host immunity and comorbidities.

## 4. COVID-19: Laboratory Abnormalities and Biomarkers—A Quick Overview

**Figure 3:**
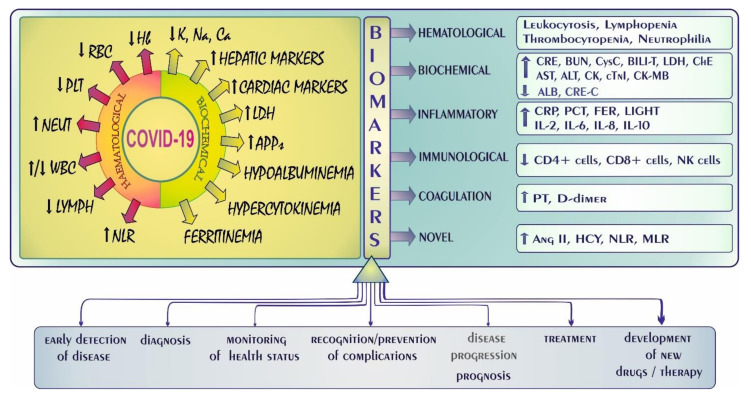
Hematological/biochemical abnormalities and biomarkers in COVID-19. Elaborated on the basis of available literature data [20,22,28,29,30,31,32,33,34,35,36,37,38]. Hb: hemoglobin; RBC: red blood cells; PLT: platelets; NEUT: neutrophils; WBC: white blood cells; LYMH: lymphocytes; NLR: neutrophil–lymphocyte ratio; APPs: acute-phase proteins; LDH: lactate dehydrogenase; K: potassium; Na: sodium; Ca: calcium; CRE: creatinine; BUN: blood urea nitrogen; CysC: cystatin C; BILI-T: total bilirubin; ChE: cholinoesterase; AST: aspartate aminotransferase; ALT: alanine aminotransferase; CK: creatine kinase; cTnI: cardiac troponin I; CK-MB: creatine kinase-myocardial band; ALB: albumin; CRE-C: creatinine clearance; CRP: C-reactive protein; PCT: procalcitonin; FER: ferritin; LIGHT: tumor necrosis factor superfamily member 14; IL: interleukin; NK cells: natural killer cells; PT: prothrombin time; Ang II: angiotensin II; HCY: homocysteine; NLR: neutrophil-lymphocyte ratio; MLR: monocyte-lymphocyte ratio. ↑ increase; ↓ decrease.

COVID-19 is characterized by several hematological and biochemical alterations. The most frequent changes in laboratory findings in COVID-19 patients (requiring hospitalization) along with biomarkers related to COVID-19 severity are summarized in Figure 3. Among hematological parameters, a lowered hemoglobin (Hb) level, reduced numbers of red and white blood cells (RBCs and WBCs), and a decrease in platelet (PLTs) and lymphocyte (LYMPHs) counts were noted. In turn, the neutrophil (NEUT) count and the neutrophil-to-lymphocyte ratio (NLR) were observed to be elevated [20,28,29]. Some of these indices, such as lymphocytopenia and thrombocytopenia, also appear at the early stage of SARS-CoV-2 infection [28]. As for biochemical indices, lowered levels of K, Na, Ca, and increased activity/levels of lactate dehydrogenase (LDH), acute-phase proteins (APPs), and hepatic/cardiac markers were recorded. Hypoalbuminemia, hypercytokinemia, and ferritinemia were also noted (Figure 3).

Undisputed is the fact that biomarkers are key indicators of biological processes (both normal and pathological) and pharmacological responses to a therapeutic intervention. It is also undeniable that a panel of biomarkers, in comparison with single indicators, may not only provide more reliable and comprehensive information but also point to the most appropriate therapeutic strategies and help to identify high-risk groups [37]. As presented in Figure 3, biomarkers play an important role in early detection of disease, identification of the stages and severity of illness, diagnosis, and monitoring of health status. They are also crucial in the recognition and prevention of complications, disease prognosis, and treatment. The role of biomarkers in the development of new drugs and therapeutic interventions is equally important.

Among the hematological markers, leukocytosis, lymphopenia, thrombocytopenia, and neutrophilia were noted. In turn, such biochemical markers as creatinine (CRE), blood urea nitrogen (BUN), cystatin C (CysC), total bilirubin (BILI-T), lactate dehydrogenase (LDH), cholinoesterase (ChE), alanine and aspartate aminotransferase (ALT and AST, respectively), creatine kinase (CK), cardiac troponin I (cTnI), and creatine kinase-myocardial band (CK-MB) were found to be elevated. In contrast, creatinine clearance (CRE-C) and albumin (ALB) were lowered. As for the inflammatory markers, C-reactive protein (CRP), procalcitonin (PCT), ferritin (FER), and tumor necrosis factor superfamily member 14 (LIGHT) as well as such interleukins as IL-2, IL-6, IL-8, and IL-10 were demonstrated to be increased. In turn, the number of certain circulating immune cells—i.e., CD4+ and CD8+ cells as well as natural killer (NK) cells—were reduced, whereas some coagulation markers such as prothrombin time (PT) and D-dimer were elevated according to [33,36]. Among the potential novel biomarkers, the homocysteine (HCY) and angiotensin II (Agn II) levels as well as the neutrophil-lymphocyte (NLR) and monocyte-lymphocyte (MLR) ratios were recorded to be higher. As suggested, the increase in these indices can be used as prognostic markers for predicting poor outcomes [33,39].

## 5. COVID-19: Therapeutic Approach—A Brief Outline

**Figure 4:**
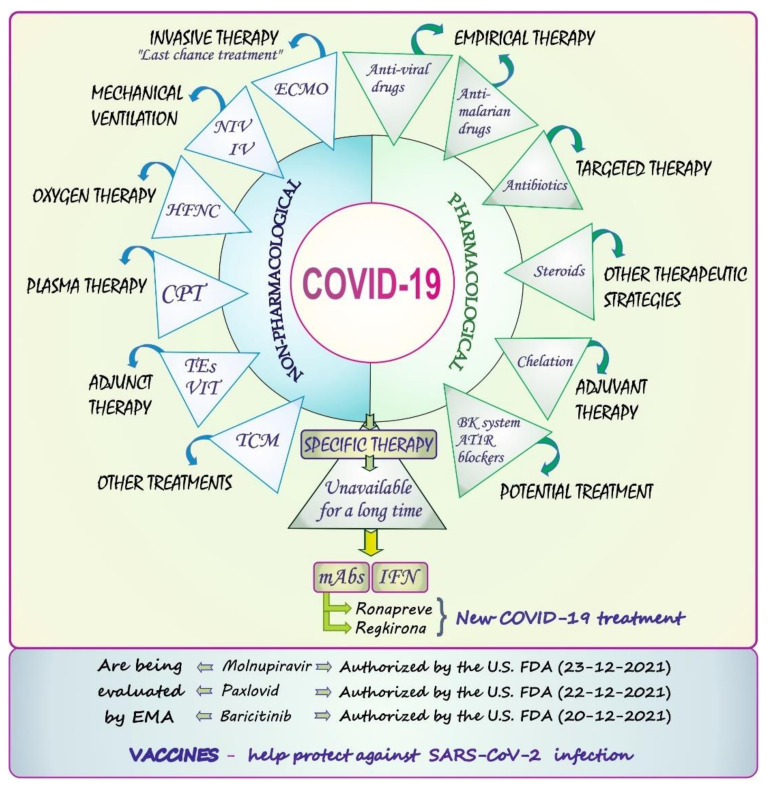
Therapeutic strategies against COVID-19. Elaborated on the basis of available literature data [20,22,32,40,41,42,43,44,45,46,47,48,49,50,51]. ECMO: extracorporeal membrane oxygenation; NIV: non-invasive ventilation; IV: invasive ventilation; HFNC: high-flow nasal cannula; TEs: trace elements; VIT: vitamins; TCM: traditional Chinese medicine; CPT: convalescent plasma therapy; BK: bradykinin; AT1R: angiotensin receptor 1; mAbs: monoclonal antibodies; IFN: interferon; EMA: European Medicines Agency; FDA: Food and Drug Administration.

The COVID-19 pandemic is a challenge for scientists to design effective therapeutic strategies for the SARS-CoV-2 infection. The pharmacological and non-pharmacological options, along with available COVID-19 specific treatment are concisely presented in Figure 4 and briefly described in the current section.

The pharmacological approach includes empirical, targeted, and adjuvant therapies with antiviral/antimalarial medications, antibiotics, and chelators, respectively. As for antiviral drugs, favipiravir, ribavirin, darunavir, and lopinavir in conjunction with ritonavir or oseltamivir as well as the ribavirin-pegylated interferon combination and remdesivir, for which a conditional recommendation against its use for the treatment of COVID-19 patients has been issued in the updated guidelines from WHO [52], are taken into account as candidate medicines [42,50]. It has also been reported that quinoline-containing antimalarial derivatives—i.e., chloroquine (CQ) and hydroxychloroquine (HCQ)—may exhibit potential in fighting SARS-CoV-2 infection [53,54]. The mechanisms of action of the above-mentioned medications are not analyzed in the present report, as they were described in detail in other papers [53,54,55]. Steroid treatment is another option, but this kind of therapy is mainly recommended for seriously and critically ill patients. According to WHO, steroids should be limited with the decreasing severity of the disease [56]. The use of iron chelators is another therapeutic strategy. Deferoxamine, for example, has been suggested to improve the clinical outcomes for COVID-19 patients and reduce the severity of COVID-19 infection [57]. Some authors highlight the high therapeutic value of Fe chelators during the COVID-19 pandemic [44], whereas others also emphasize that “…chelation should not be employed until there is evidence that elevated iron levels exist and are relevant…” and stress that before chelation is applied “…it must be determined whether COVID-19 leads to elevated iron levels or AI” [58] (p. 1). Other potential therapies are targeted at the use of the bradykinin (BK) system blockers [40] and angiotensin receptor 1 (AT1R) antagonists [43].

Monoclonal antibodies (mAbs) and interferons (IFNs) should also be mentioned as specific forms of therapy against COVID-19 [32,50]. mAbs are considered a promising approach in treatment of high-risk mild-to-moderate non-hospitalized patients. Aleem and Slenker [59] have presented a detailed discussion of the mechanism of action of mAbs against SARS-CoV-2 and clinical indications of mAbs therapy for patients who are at high risk of developing severe illness. Generally, the data show inhibition of the pathogenicity of the virus on the one hand and draw attention to several side effects that can appear during treatment on the other hand. Therefore, the need for further research to certify the SARS-CoV-2 neutralization effects and limit/mitigate the adverse events associated with this kind of therapy is emphasized [60]. As for IFN, Darazam et al. [61] evaluated the safety and efficacy of two most promising type I interferons, i.e., interferon beta-1a (IFNβ-1a) and interferon beta-1b (IFNβ-1b), on the course and outcomes of severe COVID-19 patients. The authors demonstrated reductions in time to clinical improvement induced by IFNβ-1a and numerical reductions in the case of IFNβ-1b. They concluded that, due to its excellent safety profile and possible benefits, IFNβ-1a may be a reasonable choice for patients with the severe COVID-19 form. However, the importance of IFNs in fighting the COVID-19 pandemic requires additional studies, as highlighted by researchers [40,49].

On 11 November 2021, the Committee for Medicinal Products for Human Use (CHMP) of the European Medicines Agency (EMA) delivered a favorable opinion with regard to two COVID-19 drugs—i.e., ronapreve and regkirona—which are the first mAbs against the coronavirus. The EMA recommended both preparations to be given marketing authorization in Europe [62,63]. Molnupiravir, i.e., an isopropylester prodrug of the nucleoside analog β-D-N^4^-hydroxycytidine (NHC) with a known mechanism of action [64,65], is the next important candidate in the COVID-19 treatment. The results of a Phase 2a clinical trial designed to evaluate the safety, tolerability, and antiviral activity of this drug in the treatment of COVID-19 patients revealed its antiviral efficacy and a desirable safety and tolerability profile [66]. However, since NHC has been reported to be mutagenic to mammalian cells [67], the risk for the host has been stressed to be carefully evaluated [67]. The researchers also highlight the need for further studies to assess the potential off-target effects in this case [48]. The data on the use of molnupiravir against COVID-19 are reviewed by EMA [68], which has issued advice on its use for the treatment of COVID-19 [69]. On 23 December 2021, molnupiravir was authorized by the U.S. Food and Drug Administration (FDA) for the emergency use in the treatment of mild-to-moderate COVID-19 in adults with positive SARS-CoV-2 test results who are at high risk for progressing to severe COVID-19, including hospitalization or death, and for whom alternative COVID-19 treatment options are not accessible or clinically appropriate [8]. Other drugs, such as paxlovid and baricitinib, were also authorized by the U.S. FDA on 22 December 2021 and 20 December 2021, respectively: paxlovid for the emergency use in the treatment of mild-to-moderate COVID-19 in certain adults and pediatric patients with SARS-CoV-2 infection and baricitinib for the treatment of COVID-19 hospitalized adults and pediatric patients (2 years of age or older) requiring supplemental oxygen, non-invasive or invasive mechanical ventilation, or extracorporeal membrane oxygenation (ECMO) [8]. Both drugs are being evaluated by EMA [7]. EMA also warns against overuse of COVID vaccines, as overly frequent booster doses could potentially adversely affect the immune response, and recommends increasing the booster dose interval.

The non-pharmacological activities undertaken to treat COVID-19 patients include both non-invasive and invasive ventilation (NIV and IV), which are used in mild/moderate cases and in severe COVID-19 patients, respectively [22]. A high-flow nasal cannula (HFNC), effective in the management of acute hypoxemic respiratory failure associated with COVID-19, is another non-pharmacological approach [70]. ECMO referred to as the “last chance treatment” is the next valuable option, but this strategy is applied in patients with COVID-19-related ARDS. Although its use may save lives, ECMO can intensify cytokine storms and consequently cause multiorgan failure [32]. Convalescent plasma therapy (CPT) is another therapeutic tool. As assessed by Rajendran et al. [71], this kind of intervention may improve the clinical outcome in severe disease and reduce mortality.

Literature data also point to traditional Chinese medicine (TCM) as a potential therapeutic option in treating COVID-19 patients, but further studies are needed to evaluate the efficacy and safety of TCM in the COVID-19 treatment and examine the mechanism of TCM action [72]. The possible therapeutic role of certain vitamins—mainly A, B, C, D, and E—has been highlighted as well [42,73]. For example, Jovic et al. [74] reviewed the current evidence base and critically appraised the potential immunomodulatory and antioxidant roles of vitamins A to E in the context of respiratory disease and their roles in the fight against COVID-19. The data on the doses of recommended vitamins for COVID-19 were summarized as well [50]. Some minerals such as Mg, Zn, Se, Cu, and Fe may also be helpful in the fight against coronavirus disease, as they can modulate the immune system [44,51,73,75,76,77,78,79,80]. More information about this issue is collected and concisely summarized in one of the further sections of the present review.

## 6. COVID-19: Sociodemographic/Behavioral Factors and Comorbid Conditions Affecting Disease Susceptibility, Severity, and Mortality in a Nutshell

**Figure 5:**
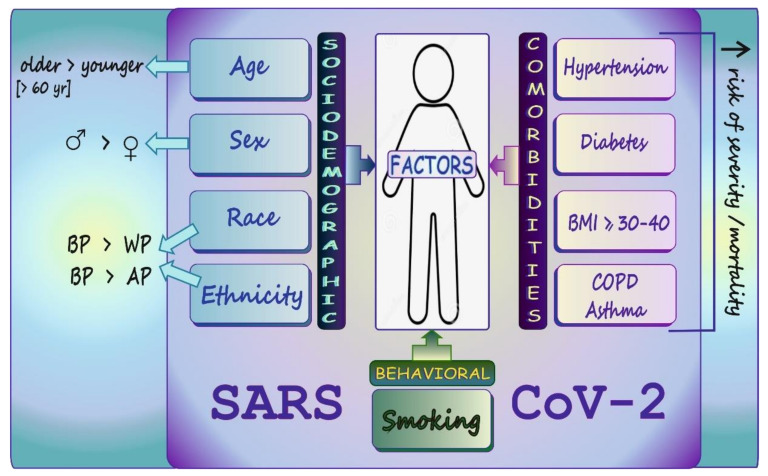
Sociodemographic/behavioral factors and comorbid conditions affecting COVID-19. Elaborated on the basis of available literature data [81,82,83]. BMI: body mass index; COPD: chronic obstructive pulmonary disease; BP: black people/patients; WP: white population; AP: Asian patients; yr: year; >: more susceptible. ↑: increase.

Since data on certain relevant factors affecting COVID-19 infection, severity, and mortality has already been published [81,82,83], only the most important aspects related to this issue are briefly summarized in Figure 5 and concisely described in the present section.

Some sociodemographic (i.e., age, sex, race, ethnicity) and behavioral factors (smoking) as well as some comorbidities may affect the course of COVID-19. Many studies have indicated that the elderly vs. the young and men vs. women are more susceptible to infection [84,85,86,87]. Some authors also found strong associations of older age, male sex, and certain comorbidities with hospital admission and risk of critical illness in COVID-19 patients [88]. Additionally, Marin et al. [89], who reviewed data on predictors of COVID-19 severity, indicated older age as a major predictor of mortality. Higher COVID-19 severity was also observed by Ebinger et al. [90] in older, male, obese, diabetic patients, and in African Americans. As for race/ethnicity, Aldridge et al. [91], who examined the risk of infection and death related to COVID-19, concluded that Black, Asian, and Minority Ethnic (BAME) people are at increased risk of death due to this illness. Gross et al. [92]—who focused on Black, Latinx, and white population—noted that Black people and the Latinx population had higher COVID-19-associated risk of death than white patients.

Hu et al. [93], who analyzed the clinical course of COVID-19 to identify risk factors associated with clinical outcomes, demonstrated that smoking is an independent risk factor for an unfavorable outcome. Umnuaypornlert et al. [94], who conducted a comprehensive systemic review and meta-analysis on the association between smoking and negative outcomes in COVID-19 patients, concluded that smoking significantly increases the risk of COVID-19 severity and patient death. They suggested that smoking cessation should be recommended for all smokers along with avoidance of secondhand smoke by non-smokers. Furthermore, Patanavanich and Glantz [95]—who studied the association between smoking and COVID-19 disease progression—stressed that smoking is an independent risk associated with severe progression of COVID-19, including mortality. They highlighted that these effects seem to be higher in young people. Similarly, Vardavas and Nikitara [96], who conducted a systemic review of studies on COVID-19 providing information on patients’ smoking status, emphasized that smoking is associated with negative progression and adverse COVID-19 outcomes.

The presence of comorbidities such as hypertension, diabetes mellitus (DM), and chronic obstructive pulmonary disease (COPD) has also been reported to be associated with a severe COVID-19 course [85,97,98]. The available data also indicate that obese patients are at increased risk of severe COVID-19 [99,100,101]. As for asthma, the analysis of clinical results performed by Izquierdo et al. [86] demonstrated that the increased risk of hospitalization due to COVID-19 in patients with this a long-term lung disease is highly associated with age and related comorbidities. As reported by Terry et al. [102], who reviewed many studies related to the prevalence of asthma in patients with COVID-19, there is no clear evidence of increased risk of COVID-19-related hospitalization, severity, or mortality due to this illness.

## 7. COVID-19: Elements

### 7.1. Impact on Immune Response—A Concise Summary

**Figure 6:**
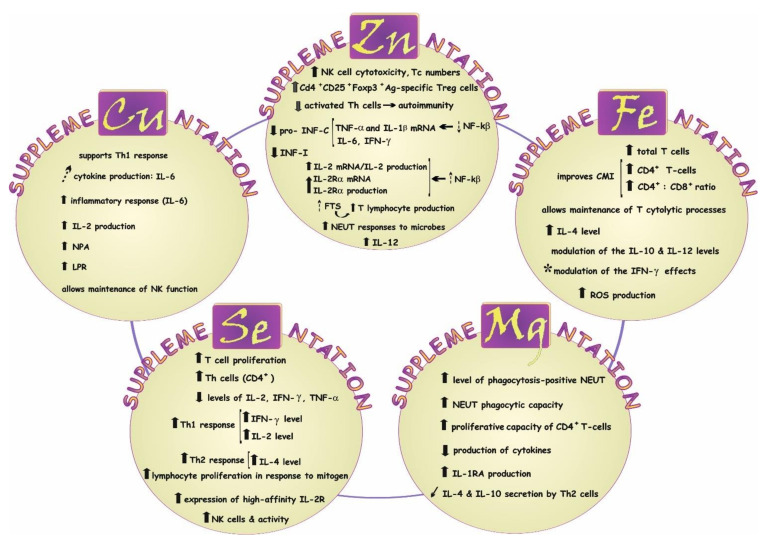
Impact of selected elements on immune responses. Elaborated on the basis of available literature data [75,111,112,113,114,115,116,117,118,119,120,121,122,123,124,125,126,127,128,129,130,131,132,133,134]. NK: natural killer; Tc: cytotoxic T cell; Treg: regulatory T cells; Ag: antigen; Foxp3+: forkhead box transcription factor; Th: helper T cell; pro-INF-C: pro-inflammatory cytokines; TNF-α: tumor necrosis factor alpha; IL-1β: interleukin 1 beta; NF-κB: nuclear factor kappa B; ROS: reactive oxygen species; IL-6: interleukin 6; IFN-γ: interferon gamma; INF-I: infection incidence; IL-2: interleukin 2; IL-2Rα: soluble IL-2 receptor alpha; IL-2R: IL-2 receptor; FTS: thymulin; CMI: cell-mediated immunity. NEUT: neutrophils; NPA: neutrophil phagocytic activity; LPR: lymphocyte proliferation response. ↑: increased; ↓: decreased; 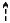: increased activity; 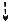: inhibited activity; 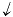: trend toward an increase; *↓*: inhibited secretion. ^*^: in vitro studies.

Such elements as Mg, Zn, Cu, Fe, and Se are well known to play a role in the regulation of immune responses [103,104,105,106,107]. Therefore, the maintenance of homeostasis of these essential minerals may be crucial for adequate immune activity and effective fight against infections [77,108,109,110]. These elements are related to both cell mediated and humoral immunity. Their immunomodulatory potentials are collected in Figure 6 and briefly summarized in this section.

#### 7.1.1. Magnesium (Mg)

Mg has been reported to elevate the level of phagocytosis-positive NEUT and NEUT phagocytic capacity, lead to an increase in the proliferative capacity of CD4+ T cells [111], and reduce cytokine production [112] by reducing nuclear factor kappa B (NF-kB) activation [135]. Mg can also stimulate the expression of anti-inflammatory markers in a dose-dependent manner [136] and the production of the IL-1 receptor antagonist (IL-1RA) [113]. Inhibition of the secretion of IL-4 and IL-10 by Th2 cells has been noted as well [114].

#### 7.1.2. Zinc (Zn)

Zn has been reported to (a) increase CD4+ and CD8+ production [137], (b) enhance NK cell cytotoxicity according to [138], (c) elevate neutrophil responses to microbes [139], (d) induce CD4+CD25+Foxp3+ antigen (Ag)-specific regulatory T (Treg) cells [134], (e) suppress interferon gamma (IFN-γ) production [134], (f) reduce the number of activated Th cells according to [75], (g) restore the activity of the Zn-dependent hormone thymulin (FTS) according to [75] involved in maturation and differentiation of T lymphocytes [140], (h) decrease infection incidence (INF-I) [125], modulate interleukin 6 (IL-6) levels [141], and (i) inhibit the induction of tumor necrosis factor alpha (TNFα) and interleukin 1 beta (IL-1β) mRNA by inhibiting the activity of NF-κB [126], which is the main regulator of proinflammatory responses [125]. Moreover, studies on HUT-78 cells conducted by Prasad et al. [118] demonstrated that Zn enhances the production and gene expression of IL-2 and interleukin-2 receptor alpha (IL-2Rα) via NF-kB activation. Additionally, Zn has been reported to cause an increase in the level of one of the critical cytokines—i.e., interleukin 12 (IL-12) [122,130]—which plays a vital role in both innate and adaptive immunity [142,143].

#### 7.1.3. Copper (Cu)

Cu increases lymphocyte proliferation response (LPR) [144], elevates neutrophil phagocytic activity (NPA) [144], supports Th1 response according to [145], and causes a significant increase in the production of interleukin 2 (IL-2) [146], which is crucial for T helper cell proliferation, NK cell cytotoxicity, and B cells function [147,148]. Cu may also enhance inflammatory response (IL-6) according to [145] and affect the level of IL-6 [149], i.e., an essential inflammatory mediator capable of exerting a protective effect during acute inflammation [150].

#### 7.1.4. Iron (Fe)

Fe has been found to increase total T cells [119], improve the CD4+ cell count and CD4:CD8 ratio [128], elevate the level of IL-4 [120], as well as modulate the IFN-γ effects [115] and the levels of IL-10 and IL-12 according to [124]. By the ability to produce free radicals, this element may also play an important role in the prevention of infection [151].

#### 7.1.5. Selenium (Se)

Se has been reported to increase T cell proliferation [121], elevate the Th cell (CD4) count [123], increase NK cells according to [127], improve NK cell activity [116], and enhance the expression of high-affinity IL-2 receptor (IL2-R) [117]. This element also contributes to an increase in Th1 and Th2 responses, i.e., elevated IFN-γ/IL-2 levels and IL-4 concentrations, respectively [152]. Se may also increase lymphocyte proliferation in response to mitogen stimulation [117] and decrease the levels of IL-2, IFN-γ, and TNF-α [131,133].

### 7.2. Blood and Urinary Levels of Elements and Studies on Mineral Supplementation

This section collects results of studies on evaluation of metal levels in COVID-19 patients (both essential elements, including such micro- and macro-elements as Fe, Zn, Cu, Se, Mn, Cr, Mg, Ca, Na, and K and the most toxic metals—i.e., Cd, Pb, and Hg) and findings of research focused on the effects of supplementation with minerals in COVID-19 subjects.

**Figure 7:**
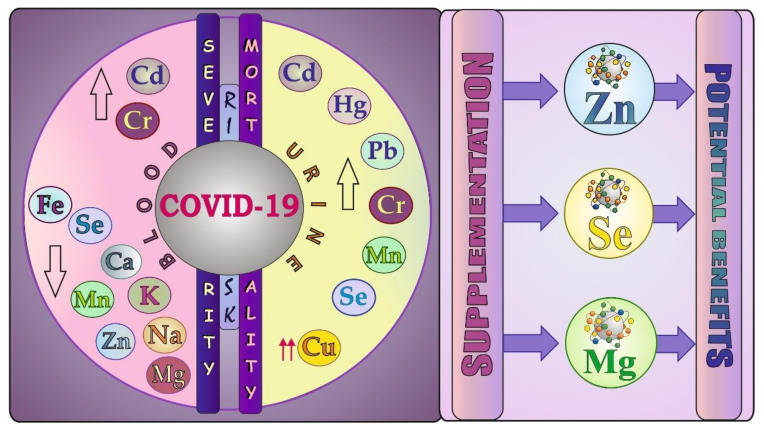
Variations in blood and urinary metal levels in association with disease severity and mortality and minerals with potential clinical significance in COVID-19. Elaborated on the basis of available literature data [38,51,153,154,155,156,157,158,159,160,161,162,163,164,165,166,167,168,169,170]. Cd: cadmium; Hg: mercury; Pb: lead; Cr: chromium; Mn: manganese; Se: selenium; Cu: copper; Fe: iron; Ca: calcium; K: potassium; Na: sodium; Mg: magnesium, Zn: zinc. ↓ decrease; ↑ increase; ↑↑ hyperincrease.

A brief summary of variations in the blood and urinary levels of elements in relation to the disease severity and mortality along with minerals used (to date) in the supplementation strategy in COVID-19 patients is presented in Figure 7. More information about this issue is collected in Tables S1 and S2 and described in detail in this part.

#### 7.2.1. Essential Trace Elements (Fe, Zn, Cu, Se, Mn, Cr)

##### Fe

The literature comprises some articles on the level of Fe in COVID-19 subjects. For example, Sonnweber et al. [35], who examined patients with mild to critical COVID-19, demonstrated an association between this illness and prolonged disturbances of Fe homeostasis. Thirty percent of all observed subjects had Fe deficiency two months after the COVID-19 onset. A significantly lowered level of this element was also found by Skalny et al. [171] in the serum of mild, moderate, and severe COVID-19 patients, compared to the control, by Zeng et al. [163] in the whole blood of patients who developed a severe form of COVID-19, in comparison with non-severe cases, and by Zhao et al. [159] in severely ill COVID-19 patients, compared to mild cases (Table S1). The serum Fe deficit was also observed to be closely correlated with COVID-19 severity and mortality and was found to be an independent risk factor for death in patients with COVID-19 [159]. Additionally, variations in the concentration of one of the markers of Fe metabolism—i.e., ferritin (FER)—in the serum of patients, depending on the COVID-19 severity were noted. For example, Alkattan et al. [157] reported that people with severe disease had a 3.5-fold higher concentration of this protein than those with non-severe COVID-19. Similarly, Sonnweber et al. [35], Yasui et al. [162], and Alkattan et al. [157] recorded a higher level of FER in the serum of severe case patients, compared to non-severe, mild/moderate group (Table S1). Taken together, these data suggest that Fe disorders may affect the COVID-19 course.

##### Zn

The literature also provides interesting data on the levels of Zn in patients with COVID-19. Abdelmaksoud et al. [167], who assessed the Zn levels in the serum of COVID-19 patients with different grades of severity, did not reveal any significant differences in the concentration of this element between patient subgroups. Similarly, Alkattan et al. [157] did not observe significant changes in the level of Zn in the serum of severe versus non-severe COVID-19 patients (Table S1). In turn, Muhammad et al. [172], Elham et al. [173], and Jothimani et al. [38] reported a significant reduction in the concentration of Zn in the plasma/serum of COVID-19 patients, in comparison with the control. It was also reported by Dubourg et al. [158] in the plasma of patients with poor versus good clinical outcome and by Zeng et al. [163] in the whole blood of severe versus non-severe COVID-19 patients (Table S1). In addition, a study conducted by Jothimani et al. [38] revealed that 57.4% of COVID-19 subjects were Zn deficient and developed more complications. As observed, the deficit of Zn was associated with a prolonged hospital stay and increased mortality. Skalny et al. [171], who showed a significantly lowered Zn concentration in the serum of moderate and severely ill COVID-19 patients compared to the control, also observed a gradual decrease in the level of this metal with increasing severity of this illness. Moreover, Heller et al. [80] demonstrated that Zn concentrations in COVID-19 patients were lower than in healthy subjects and found that the majority of samples from the non-survivors (i.e., 73.5%) and almost half of the samples from the survivors (i.e., 40.9%) were below the threshold for Zn deficiency (i.e., <638.7 μg/L) (Table S1). Pour et al. [174] also indicated that the serum Zn concentration in the deceased group was significantly lower, compared to the recovered group (Table S1). Finally, Yasui et al. [162], who evaluated the predictive factors for a critical illness of COVID-19, indicated a remarkably lower concentration of this element in the serum of severe patients (Table S1, [35,38,51,80,157,158,159,162,163,164,165,167,168,171,172,173,174,175,176]) and suggested that prolonged hypozincemia (<70 μg/dL) can be a risk factor for a severe COVID-19 course.

To sum up, the majority of the above-mentioned studies demonstrated that COVID-19 is related to a significant fall in the level of Zn, which seems to point to the need of supplementation with this essential element of COVID-19 subjects to reduce poor outcomes. The analysis of available literature data showed single studies on supplementation with this mineral in COVID-19 patients. One of them—i.e., a prospective clinical trial study—provides results of the serum Zn levels in subjects with COVID-19 of various severities (with and without olfaction dysfunction) and data on the effect of Zn therapy in the recovery of smell disorders [167]. The second study presents findings from a retrospective design on the serum Zn and Cu levels and Zn supplementation in parenteral nutrition (PN) as well as their association with inflammatory markers and prognosis [168]. The third article shows data on the clinical significance of Zn and Se in critically ill COVID-19 patients with severe ARDS [169]. The fourth study presents the results of a retrospective observational design on administration of Zn with HCQ and azithromycin (AZM) in hospitalized COVID-19 patients [177]. The fifth article shows findings on the association between Zn and the survival of hospitalized patients with COVID-19 [178]. The sixth paper presents results of a retrospective case series study on the treatment of COVID-19 outpatients with Zn in combination with low-dose HCQ and AZM [179] and another two studies provide data on the efficacy and safety of Zn as adjunctive therapy in critically ill patients with COVID-19 [180] as well as the effectiveness of this element in the prevention and mitigation of COVID-19 [181]. Finally, the last multi-center cohort study shows data on the effect of Zn with a ionophore on COVID-19 in-hospital mortality rates [182].

The first study mentioned above [167] (including 134 participants, 105 with olfactory disturbances) showed a significantly lowered duration of recovery of olfactory function in COVID-19 patients receiving Zn therapy (49), in comparison with those without Zn treatment (56). However, no marked differences in the total recovery duration from COVID-19 between both groups were noted. The second study [168] (including 35 participants, aged ~65 y) demonstrated that the serum Zn level during NP support is inversely correlated with the length of hospital stay but not with mortality, and this association was observed in patients in both ICU and intermediate care. The third one, including 22 patients (median age 60.5 y), showed a beneficial effect of supplementation with Zn and Se in the treatment of critically ill COVID-19 patients [169]. The fourth study [177] with 932 subjects (~63 y of age), with 411 receiving Zn together with HCQ and AZM, showed a reduced risk of mechanical ventilation and mortality in subjects receiving Zn along with HCQ and AZM, in comparison with those without Zn supplementation. The fifth study mentioned above [178] including 242 patients (median age 65 y), with 196 administered zinc sulfate at a total daily dose of 440 mg (100 mg elemental Zn), did not reveal any significant beneficial effect of the supplementation with this element. The sixth study with 141 COVID-19 patients (median age 58 y) receiving Zn, HCQ, and AZM (i.e., Zn sulfate: 220 mg, 50 mg elemental Zn, once daily; HCQ: 220 mg twice daily; AZM: 500 mg once daily) for five consecutive days demonstrated that the treatment with the triple therapy may prevent a large number of hospitalizations [179]. One of three last studies—i.e., a two-center retrospective study [180] on 164 patients aged ≥ 18 y (82 receiving Zn at a daily dose of 220 mg, 50 mg elemental Zn)—indicated that Zn, as an additional treatment, may have survival benefits. Other interventional, prospective, single-blind study [181] involving 96 control subjects (71 y of age) and 104 patients (74 y of age) treated with 10, 25, or 50 mg of Zn picolinate daily revealed that the Zn supplementation in all three doses may be effective prophylaxis of symptomatic COVID-19 and may mitigate the severity of COVID-19 infection. The last one including 3473 adult hospitalized patients showed a significant reduction in hospital mortality rates after administration of Zn in combination with ionophore [182].

##### Cu

The level of Cu in COVID-19 patients was also evaluated providing the data presented below. A significant decrease in the concentration of this element was noted by Muhammad et al. [172] in the plasma of COVID-19 patients, in comparison with the control group, by Arrieta et al. [168] in critically ill COVID-19 patients, and by Hackler et al. [175] in non-surviving versus surviving COVID-19 subjects (Table S1). The latter authors [175], who assessed the potential value of the biomarkers of the Cu and Se status, demonstrated that a composite biomarker of Cu and selenoprotein P (Se-P) may provide reliable information on the COVID-19 course and survival odds. In turn, a significant increase in the serum Cu concentration was noted by Skalny et al. [171] in patients with moderate and severe COVID-19 groups, in comparison with the control, and by Zeng et al. [163] in the whole blood of severe versus non-severe COVID-19 patients. Finally, Alkattan et al. [157] did not report any significant changes in the serum Cu concentration in severe COVID-19 patients, compared to non-severe individuals (Table S1).

A deficit of Cu in the blood of critically ill COVID-19 patients reported by some authors cited above may suggest the need for supplementation with this mineral. Unfortunately, the lack of findings from observational studies/clinical trials on the evaluation of the potential efficacy of supplementation with these essential elements in severe COVID-19 patients does not allow us to discuss the results more extensively. On the other hand, the elevated level of this mineral in the blood of COVID-19 patients reported by other research groups and the lack of changes in its concentration noted by Alkattan et al. [157] makes Cu supplementation not so obvious.

The findings obtained by another research group [164] focused on analysis of urinary concentrations of essential and/or toxic metals and on the associations between them and severe illness/outcome in patients with COVID-19 showed a significant increase in the level of this element in the urine of severe COVID-19 patients, in comparison with non-severe cases. The creatinine-adjusted urinary concentration of Cu was also significantly elevated in severe patients, compared to non-severe subjects (Table S1). Additionally, the level of Cu in the urine of severe patients was significantly higher in the deceased group than in the recovered group and, when adjusted by urinary creatinine, its concentration still remained markedly elevated in the deceased versus recovered group [164]. As suggested, high urinary Cu excretion can be associated with liver dysfunction and enhanced inflammatory processes. The authors also showed that the urinary creatinine-adjusted Cu concentration (>25.57 µg/g and >99.32 µg/g) was associated with significantly increased risk of severe illness and fatal outcome, which suggests that the urinary Cu may serve as a prognostic factor of severe COVID-19.

##### Se

Certain studies aimed at analyzing the level of Se in COVID-19 patients also revealed disturbances in homeostasis of this mineral. For example, a markedly elevated concentration of Se was found by Alkattan et al. [157] in the serum of severe versus non-severe COVID-19 patients. In turn, a significant decrease in the concentration of this element was noted by Majeed et al. [176] and Muhammad et al. [172] in the serum/plasma of COVID-19 patients, in comparison with the control. A significantly reduced serum Se level was also found by Jahromi et al. [165] in patients with severe COVID-19, in comparison with those classified into a mild and moderate group, and by Skalny et al. [171] in the serum of moderate and severely ill COVID-19 subjects, compared to the control (Table S1). The latter authors also observed a gradual decrease in the level of Se in the serum with increasing COVID-19 severity. A significant decline in the concentration of Se and, additionally, Se-P was recorded by Moghaddam et al. [51] in the serum of non-survivors, in comparison with survivors (Table S1). The authors also found a strong positive correlation between the Se and SeP levels in the group of non-survivors and survivors.

In addition to the above-mentioned findings, Zeng et al. [164] recorded a significant decrease in the level of Se in the urine of severe versus non-severe COVID-19 patients, and a significant increase in its urinary concentration after creatinine adjustment (Table S1). Additionally, among the severe cases, the creatinine-adjusted urinary level of this element was markedly elevated in the deceased than recovered group [164]. As suggested, liver dysfunction may be responsible for such an effect according to [164].

To the best of our knowledge, there is only one paper presenting the findings of an observational study on the influence of administration of Se in COVID-19 ICU patients with severe ARDS [169], in which 22 subjects (~60.5 y of age) received 1.0 mg of intravenous Se (as selenite) per day for 2 weeks and artificial diet additionally containing Se and Zn. As shown, the selected supplementation strategy effectively compensated Se and Zn deficiency as well as Se-P deficit in COVID-19 patients. It was also found that Se-P inversely correlated with CRP, IL-6, IL-1β, and IL-10 and positively correlated with CD8+ T cells and NK cells. Additionally, Se was negatively correlated with CRP and positively associated with the number of NK cells. Thus, the results of this study provide tangible evidence for the importance of Se and Zn supplementation in severe COVID-19 patients.

##### Mn and Cr

The levels of Mn and Cr, which may act as anti-inflammatory agents [183,184], were also investigated in COVID-19 patients. For example, significantly lowered concentrations of Mn were noted by Muhammad et al. [172] in the plasma of COVID-19 patients, compared to control subjects and by Zeng et al. [163] in the whole blood of severe, compared to the non-severe group. The latter authors [163] also found significantly elevated concentrations of Cr in the whole blood of severe COVID-19 patients, compared to non-severe cases (Table S1), and in the whole blood of deceased versus recovered patients. In addition, they found markedly elevated concentrations of Mn in the urine of patients with severe COVID-19 (both without and after urinary creatinine adjustment), in comparison with non-severe subjects (Table S1), and in the urine of the deceased group (without and after urinary creatinine adjustment), compared to recovered ones. The urinary excretion of Cr also markedly increased in severe versus non-severe cases and its concentration (without and after urinary creatinine adjustment) in the severe patients was significantly higher in the deceased than recovered group [164]. As suggested, the disturbances in urinary concentrations of Mn and Cr are associated with severe illness and a fatal COVID-19 outcome.

#### 7.2.2. The Most Toxic Trace Metals (Hg, Pb, Cd)

The levels of Hg, Pb, and Cd, which are well known to be toxic to mammals, were also investigated. The reviewed data did not reveal any significant changes in the concentrations of Hg and Pb in the whole blood of severely ill and deceased patients, in comparison with the non-severe and recovered group, respectively [163]. In turn, their urinary concentrations (when adjusted by urinary creatinine) significantly increased in severe COVID-19 patients, compared to non-severe subjects (Table S1), and in the deceased versus recovered group after or without adjustment by urinary creatinine [164]. In turn, the concentration of Cd in the whole blood of severe COVID-19 patients did not change significantly, compared to non-severe subjects (Table S1), but its level significantly increased in the deceased patients, compared to the recovered group [163]. A significant increase in the concentration of this metal was also found in the urine (without and after urinary creatinine adjustment) of severe patients with COVID-19, in comparison with non-severely ill individuals, and in the deceased versus recovered group [164]. As suggested, the markedly higher Cd, Hg, and Pb levels in the urine of severe COVID-19 patients may have resulted from kidney damage.

#### 7.2.3. Macroelements (Mg, Ca, Na, K, Cl)

Given the importance of macroelements in human health [185], their inadequate balance can lead to serious health consequences and impair immune function. Some published studies focused on macroelements in COVID-19 patients revealed alterations in the Mg, Ca, Na, K, and Cl levels. For example, Quilliot et al. [186], who evaluated the prevalence of dysmagnesemia in COVID-19 patients, but not the relationship between magnesemia and patient prognosis, noted a significant decrease in the concentration of Mg in moderately versus critically ill subjects. They also found that hypomagnesemia was significantly higher in patients classified into the moderate group than in critically ill subjects, whereas the prevalence of high-level serum Mg concentrations was markedly elevated in critically ill COVID-19 patients [186]. A significant decrease in the level of Mg was also noted by Zeng et al. [163] in the blood of severe COVID-19 patients and by Alamdari et al. [187] in the blood of expired versus discharged patients (Table S2).

To date, the results of only one observational study evaluating the effect of supplementation with Mg in combination with vitamin D and B_12_ on the progression to severe outcomes in older patients (≥50 years of age) with COVID-19 [170] provided evidence of the beneficial effects of the strategy of supplementation with this mineral. As indicated by the study, the combined administration of Mg with vitamin D and B_12_ is associated with a significant reduction in the proportion of patients with clinical deterioration.

As for Ca, a markedly lowered level of this element was noted by Elham et al. [173] in the serum of COVID-19 patients versus healthy individuals and by Alkattan et al. [157] and Qian et al. [188] in the serum of severe patients versus non-severe ones. Cappellini et al. [147] also found a significant decrease in the concentration of both total Ca in the serum and ionized Ca (Ca^2+^) in the whole blood of COVID-19 subjects, compared to healthy subjects. Sun et al. [166], who examined correlations between the level of Ca in the serum and clinical outcomes in patients with SARS-CoV-2 infection, observed an association between the serum Ca concentration and the disease severity and prognosis; subjects at serum Ca levels less than 2.0 mmol/L had higher MODS incidences, septic shock, and a higher 28-day mortality rate. The incidence of hypocalcemia was found to reach 74%. Bennouar et al. [189] also demonstrated a high frequency of hypocalcemia in severe COVID-19 patients and provided evidence of their potential link to poor short-term prognosis. The authors found that only 35.8% of patients had an adequate serum Ca level and that the concentration of this element in the serum lower than 2.05 mmol/L can predict short-term mortality with a sensitivity of 84% and a specificity of 60%. The above-mentioned findings seem to point to correction of hypocalcemia, common in severely ill COVID-19 patients, to attenuate disease severity. However, in literature, there are no available data on the effects of Ca supplementation in hypocalcemic COVID-19 patients. In turn, other data (referring to other cases of critical illness) have shown that administration of Ca brings no benefit and may even be harmful [190,191]. This shows that further studies are needed to verify the beneficial and unfavorable effects of Ca supplementation in critically ill patients. In another study [163], a significantly elevated Ca concentration in the whole blood of severe COVID-19 subjects compared to non-severely ill individuals was found.

As far as Na is concerned, Alkattan et al. [157] did not observe any significant changes in the level of this element in the serum of severe versus non-severe COVID-19 patients, but other studies showed a significant reduction in the concentration of Na in the serum/plasma of patients with a severe form of COVID-19, in comparison with a group with mild disease [188,192] (Table S2). Sjöström et al. [193], who explored the dynamics of electrolytes in COVID-19 patients, revealed the presence of hyponatremia in subjects at admission followed by the development of hypernatremia during the first 2 weeks of hospitalization. As observed, hypernatremia was common and associated with a more severe COVID-19 course and a higher mortality rate. Hyponatremia, as well as hypokalemia and hypochloremia, were also recorded more frequently in patients infected with COVID-19 than in controls [194]. Moreover, hyponatremia was found to be associated with COVID-19 in patients requiring ICU admission. Gálvez-Barrón et al. [195], who analyzed the most important prognostic factors of severe disease and mortality in a cohort of oldest-old people with COVID-19 ≥ 80 years of age, demonstrated that the serum Na level was associated with mortality in these subjects. As Na deficiency may increase the risk of developing severe and fatal COVID-19 infection [196,197], the monitoring of the level of this element in severe COVID-19 seems be desirable. A significant decrease was also found in the concentration of K in the blood of deceased patients [187], in the plasma of severe and critically ill patients [155], and in the serum of non-critically ill subjects with confirmed COVID-19 [198]. In turn, Alkattan et al. [157] did not observe any significant alterations in the level of K in the serum of patients with severe versus non-severe COVID-19 subjects (Table S2, [154,155,157,163,173,186,187,188,189,192,198,199]). Finally, in severe COVID-19 patients, compared to non-severe cases, a markedly lowered concentration of Cl was also noted [157] (Table S2). These data suggest that derangements in homeostasis of the above-mentioned elements may predispose patients to severe COVID-19 symptoms.

### 7.3. COVID-19: Clinical Trials on Supplementation with Minerals—A Brief Update in a Nutshell

This section is an attempt to provide an overview of interventional clinical studies with such minerals as Zn, Cu, Se, and Mg (alone or in combination with some drugs, vitamins, or herbal formulations/plant extract), which are currently being used in COVID-19 patients or in subjects at higher COVID-19 risk. The results obtained from these clinical trials conducted in the USA, Australia, China, India, Brazil, and other countries may provide real clinical data for COVID-19 challenges and shed light on the potential suitability of minerals as prophylactic agents against SARS-CoV-2 infection. Noteworthy, due to their potent antiviral activities, these elements may be part of a preventive/therapeutic regime against SARS-CoV-2 infection. A brief summary of the current status of clinical trials with Zn, Cu, Se, and Mg, at the time of writing this review, is provided below, while more details are given in specific sections included in this part of the review.

As on 18 January 2022, one (1), seven (7), one (1), and three (3) clinical trials marked as ‘Not yet recruiting’/‘Active, not recruiting’ include Mg, Zn, Se, and Cu, respectively, one (1) and eleven (11) clinical studies with the ‘Recruiting’ status include Mg and Zn, respectively, and seven (7), two (2), and five (5) marked as ‘Recruitment complete’ include Zn, Mg, and Se, respectively.

#### 7.3.1. Zn

Clinical trials marked as ‘Not yet recruiting’/’Active, not recruiting’ (Table S3) are undertaken to evaluate (a) the efficacy of HCQ and Zn in the prevention of COVID-19 infection in military healthcare workers (NCT04377646); (b) the effect of combined supplementation with Zn and green tea extract (GTE) on reduction in symptom duration and severity from cold and flu-like illness, including COVID-19, in adult patients (NCT04898023); (c) the efficacy and safety of HCQ, AZM, and Zn in the treatment of patients with SARS-CoV-2 infection in Senegal (PACTR202005622389003); (d) the effect of Zn and ascorbic acid (AscA) supplementation in hospitalized COVID-19 positive patients in Bangabandhu Sheikh Mujib Medical University—BSMMU (NCT04558424); (e) the therapeutic role of Zn in COVID-19 patients (CTRI/2020/07/026340); (f) the efficacy of combined therapy with ivermectin (IVM), doxycycline (DOXY), Zn, vitamin D3, and vitamin C in COVID-19 infection treatment (NCT04482686); and (g) the effect of resveratrol (RSV)-assisted Zn therapy in non-hospitalized patients with SARS-CoV-2 infection (NCT04542993).

In turn, the clinical trials marked as ‘Recruiting’ (Table S3) are focused on the evaluation of (a) the effectiveness of quadruple therapy with Zn on the clinical outcomes of patients infected with COVID-19 (NCT04468139); (b) the effect of supplementation with Zn on the clinical efficacy of CQ in treatment of COVID-19 (NCT04447534); (c) the effect of ivermectin (IVM) with or without Zn in treating the COVID-19 patients (NCT04472585); (d) the effectiveness of high-dose intravenous Zn in COVID-19 positive critically ill patients (ACTRN126200000454976); (e) the efficacy and safety of therapy including AZM, HCQ, Zn, vitamin D3/B12 with or without vitamin C (vitamin C) in participants with COVID-19 (NCT04395768); (f) the efficacy of quintuple therapy (HCQ, AZM, vitamin C, vitamin D, and Zn) in the treatment of patients with COVID-19 infection (NCT04334512); (g) the effectiveness of a variety of non-prescription approaches for the treatment of non-hospitalized adults positive for COVID-19 (NCT04621149); (h) the effect of vitamin D and Zn supplementation on improving COVID-19 treatment outcomes among COVID-19 patients in India (NCT04641195); and (g) the treatment with HCQ, vitamin C, and vitamin D in combination with Zn for the prevention of COVID-19 infection in medical workers, who are at elevated risk of COVID-19 due to the exposure to COVID-19 positive patients in the Emergency Department or Intensive Care Unit (NCT04335084); as well as (h) with HCQ or IVM in combination with Zn as a prophylaxis for asymptomatic healthcare workers (NCT04384458). In the last clinical trial NCT04323228 with the ‘Recruiting’ status, a combination of dietary supplements including Zn will be used as an intervention against COVID-19.

Finally, the clinical trials marked as ‘Recruitment complete’ (Table S3) were undertaken with the aim to evaluate (a) the safety and efficacy of HCQ and Zn in combination with either AZM or DOXY in a higher risk COVID-19 positive outpatient population (NCT04370782); (b) the effect of Zn on treatment and clinical course of SARS-CoV-2 patients (IRCT20180425039414N2); (c) the impact of AscA and zinc gluconate (ZnGLU) in reducing duration of symptoms in patients diagnosed with coronavirus disease 2019 (NCT04342728); (d) the efficacy of Zn in a higher risk COVID-19 positive outpatient population (NCT04621461); (e) the effectiveness of Kabasura Kudineer (KSK) and vitamin C-Zn in the management of asymptomatic COVID-19 patients (CTRI/2020/05/025215); (f) the efficacy of prophylaxis treatment among migrant workers at high-risk of COVID-19 (NCT04446104); and (g) the efficacy of a combination of doxycycline (DOXY) and Zn in the primary prevention of COVID-19 infection in health care professionals (NCT04584567). More details related to these studies are provided in Table S3.

#### 7.3.2. Cu and Se

As far as Cu is concerned, in clinical trials marked as ‘Not yet recruiting’ only, patients with pneumonia due to SARS-CoV-2 (CTRI/2020/05/025337—phase II), asymptomatic or mildly symptomatic patients with SARS-CoV-2 infection (CTRI/2020/05/025336—phase III), and SARS-CoV-2 positive cancer patients, who are symptomatic or have COVID-19-induced pneumonia (CTRI/2020/07/026514) will be tested with a new molecule—i.e., resveratrol (RSV)—in combination with Cu in the form of RSV-Cu tablets. Table S4 provides more details about the patients and treatment.

As for Se, clinical trial with the ‘Not yet recruiting’ status (NCT04869579) will consist in the use of selenious acid (SeA) in moderately-, severely-, and critically-ill COVID-19 patients. More precisely: the COVID-19 patients will receive a SeA infusion (2000 µg) on day one and next a continuous infusion of SeA will be applied at a maintenance dose of 1000 µg daily on days 2–14 together with continued standard of care therapy (Table S5). As highlighted, “the working hypothesis of this trial is that selenium treatment would decrease the death rates and increase the rate of hospital discharges among hospitalized patients”.

Another interventional clinical studies employing this element, marked as ‘Recruitment complete’ (Table S5), were undertaken with the aim to examine (a) the effect of supplementation with Se on inflammatory markers and blood cells in COVID-19 patients (IRCT20210427051100N1); (b) the effect of administration of Se on the physical burden linked to ARDS, mortality, and the need for hospitalization in patients diagnosed with COVID-19 at early stages (IRCT20190418043307N1); (c) the increase in the chance of recovery and reduction of death rates and the need for intensive care/mechanical ventilation by adding injectable Se to the treatment regimen for 19-year-old inpatients (IRCT20160706028815N5); (d) the effect of the combination of Se, vitamin C, and methylprednisolone (MPS) on the mortality and morbidity of COVID-19 patients (IRCT20190312043030N2); and evaluate (e) the effectiveness of Se added to intravenous nutrition therapy on mortality and duration of ICU hospitalization in COVID-19 patients (IRCT20160919029870N3).

#### 7.3.3. Mg

One clinical trial with the ‘Not yet recruiting’ status is aimed to compare the safety and efficacy of vitamin D with Mg in mild to moderate COVID-19 patients (CTRI/2020/06/026189). Another clinical study, marked as ‘Recruiting’, is being done to determine whether administration of oral Mg citrate (MgCIT) and a probiotic will improve the outcome of adults hospitalized with COVID-19 (NCT04941703). In turn, clinical trials marked as ‘Recruitment complete’ were undertaken to evaluate the efficacy and safety of inhaled Mg sulfate (MgS) in combination with standard treatment in COVID-19 patients (IRCT20191211045691N1) and the effect of supplementation with vitamin D and Mg on clinical symptoms, inflammatory markers, and oxidative stress in patients with COVID-19 (IRCT20210702051763N1) (Table S6).

To date, the results of only three clinical trials—i.e., NCT04342728, CTRI/2020/05/025215, and NCT04447534 (Table S3)—have been published. The findings of the other clinical trials are still awaited.

The data of the randomized NCT04342728 clinical trial where 214 patients with SARS-CoV-2 infection received either 10-day therapy with ZnGLU (50 mg) and AscA (8000 mg) separately (58 and 48 subjects, respectively) and in combination (58 individuals) or standard care (50 subjects) showed that the treatment with these agents administered alone or together did not significantly reduce the duration of symptoms, compared to the standard care [200]. Patients without the supplementation achieved a 50% reduction of symptoms with a mean of 6.7 days, in comparison with 5.5 for AscA, 5.9 for ZnGLU, and 5.5 for AscA+ZnGLU.

In turn, the results of the prospective, single-center, open-labeled, randomized, controlled CTRI/2020/05/025215 trial with 60 asymptomatic COVID-19 patients receiving KSK (60 mL twice daily for 7 days; 30 subjects) or standard care (30 individuals), i.e., vitamin C (60000 IU, 7 days) and Zn (100 mg, 7 days) revealed a reduced SARS-CoV-2 viral load on the seventh day in both groups, with more pronounced results in the KSK group. Simultaneously, no adverse effects were observed [201].

Finally, the findings obtained from the last randomized NCT04447534 clinical trial including 191 patients with a confirmed diagnosis of COVID-19 infection receiving HCQ in combination with Zn (96 subjects, ~43 years of age) or HCQ alone (95 individuals, ~43 years of age) showed that Zn did not enhance the clinical efficacy of HCQ [202], which works as Zn ionophore, thereby increasing Zn concentration [203].

To sum up, although the results of the above-mentioned trials did not show a significant effect of Zn, other clinical trials employing this element in COVID-19 may shed different light on Zn supplementation and offer valuable data on Zn adjunct treatment. It should be added that it will be possible to discuss thoroughly the clinical value of supplementation with this mineral when the data of the trials collected in this review become available. It should also be mentioned that one of the clinical studies, observational in nature (including 229 participants), which was undertaken to evaluate the Zn, Cu, Se, vitamin A, D, and E status in elderly subjects affected by COVID-19 and to correlate this status with prognosis of this illness (NCT04877509, France), may provide interesting data on association of Zn and other micronutrients with adverse outcomes during viral infection and provide the basis for future research.

## 8. Summary

The studies on elements in COVID-19 patients overviewed in the current report showed abnormal levels of both toxic metals and those crucial for the proper function of the organism in the blood/urine of COVID-19 patients. They also revealed that, in some cases, the changes in the concentrations of certain elements are more exacerbated in subjects who developed a severe form of COVID-19, compared to those with non-severe/mild-to-moderate COVID-19 infection, and in non-survivors than in survivors. In certain cases, a gradual decrease in the levels of elements that are able to maintain an optimal immune response and effectively counteract SARS-CoV-2 infection was also observed with increasing COVID-19 severity.

Based on the presented findings, it can be concluded that SARS-CoV-2 infection interferes with the levels of metals and that variations in their concentrations can be associated with COVID-19 severity. Since this disease may have an impact on the homeostasis of some essential elements (macro-/micro-elements), the control of their levels in the blood/urine of COVID-19 patients seems to be necessary for detection of potentially imbalanced mineral homeostasis, identification more severe patients, progression of the illness, and improvement of treatment. The results collected also indicate that some elements may be important for identification of patients with a severe COVID-19 course/high mortality risk and provide convincing reasons to believe that supplementation with such minerals as Mg, Zn, and/or Se may be effective in the treatment of COVID-19 patients. As shown in some studies, supplementation with these essential elements (i.e., Mg, Zn, and/or Se) can improve poor outcomes caused by SARS-CoV-2 infection and reduce the duration of recovery of some less-severe COVID-19 related symptoms (Zn).

It should, however, be highlighted that the overviewed results were mainly obtained from observational studies, including such limitations as the variety of populations, the small-scale sample size, different measurement techniques, and different reference values. All these limitations and the lack of data from larger-scale studies, which would verify the present results to some extent, as well as the lack of results from most clinical trials on the effectiveness of supplementation of COVID-19 patients with such minerals as Mg, Zn, or Se alone or in combination with other agents (which could help to determine their role in the COVID-19 course) make it impossible to provide clear answers to the questions included in the Introduction at this stage, and this is the weakness of this review.

A strength of the review is the compilation of knowledge of the levels of some essential/toxic elements in mild, moderate, severe, and critically ill COVID-19 patients and the recent data on the effects of supplementation with some micronutrients in COVID-19 subjects, which may lay the groundwork for new therapeutic advances and further research on laboratory biomarkers for COVID-19. It may also be helpful in improving screening patients at risk and accurate diagnosis.

## 9. Conclusions

At this stage, the main conclusion that may be drawn from the overviewed data is that essential elements involved in the modulation of inflammatory processes, especially Mg, Zn, and Se, should be routinely monitored in COVID-19 patients to detect possible variations in their levels and restore their homeostasis, as they can potentially limit the development of complications and increase the chance for survival. Some of the results presented in this review clearly revealed the benefits of supplementation with these minerals, which suggests that they can be used therapeutically in severely ill COVID-19 patients. However, further well-controlled and designed studies are needed for more precise evaluation of the mineral adjunct therapy for COVID-19 and the metal levels in the context of markers of disease severity and prognosis. More studies are also necessary on composite biomarkers, which could contribute to a better approach to COVID-19 diagnosis, prognosis, and treatment. We believe that the information provided in the review will be useful not only for those interested in the role of elements in COVID-19 and therapeutic strategies against this illness, but also to anyone interested in the SARS-CoV-2 pandemic in general.

## Data Availability

Data sharing not applicable.

## Supplementary Materials

The following supporting information can be downloaded at: www.mdpi.com/article/10.3390/biology11020215/s1, Table S1: Summary of results for essential and toxic elements: COVID-19 patients. Table S2: Summary of results for macroelements: COVID-19 patients. Table S3: Summary of clinical trials with zinc (Zn) for COVID-19. Table S4: Summary of clinical trials with copper (Cu) for COVID-19. Table S5: Summary of clinical trials with selenium (Se) for COVID-19. Table S6: Summary of clinical trials with magnesium (Mg) for COVID-19.
