# Supplementary material for: Elements and COVID-19: A Comprehensive Overview of Studies on Their Blood/Urinary Levels and Supplementation with an Update on Clinical Trials
Source: Biology (Basel). 2022 Jan 28;11(2):215. doi: 10.3390/biology11020215 (PMC8869171; doi:10.3390/biology11020215)
Supplement: Supplementary file 1 [file biology-11-00215-s001.zip › biology-1531551-supplementary.pdf]

Table S1. Summary of results for essential and toxic elements: COVID-19 patients

Element	Patients (disease status)	Region	Number Sex	Age (mean/*median*/range)	MeCon (mean/*median*)*	Samples	Normal values**	Unit	Ref.
**Fe**									
	Control	Moscow, Russia	n = 43, 27 (♂), 16 (♀)	55.67	1.87	Serum	n/a	µg/mL	[171]
	Mild		n = 50, 25 (♂), 25 (♀)	50.47	1.32	-//-		-//-	
	Moderate		n = 50, 31 (♂), 19 (♀)	54.22	1.34	-//-		-//-	
	Severe		n = 50, 25 (♂), 25 (♀)	64.5	1.33	-//-		-//-	
	Mild	n/a	n = 22	58	18	Serum	n/a	µmol/L	[35]
	Moderate		n = 34		16	-//-		-//-	
	Severe		n = 53		15	-//-		-//-	
	Non-severe	Wuhan, Hubei	n = 202, 88 (♂), 114 (♀)	*69*	436.8	WB	406.5- 576.7	mg/L	[163]
	Severe	Province, China	n = 104, 60 (♂), 44 (♀)	*58*	370.24	-//-		-//-	
	Mild		n = 19	*49 (36-65)*	6.6	Serum	7.8-32.3	µmol/L	[159]
	Severe		n = 18	*55 (46-63)*	4.9	-//-		-//-	
	Critical		n = 13	*66 (56-74)*	5.2	-//-		-//-	
Fe as FER									
	Mild	n/a	n = 22	58	139	Serum	n/a	µg/L	[35]
	Moderate		n = 34		260	-//-		-//-	
	Severe		n = 53		317	-//-		-//-	
	Non-severe	Riyadh, Saudi Arabia	n = 45	47.3	331.24	Serum	12-300	ng/mL	[157]
	Severe		n = 35	58.2	1174.95	-//-		-//-	
	Mild / Moderate	Sakai, Osaka, Japan	n = 22, 12 (♂), 10 (♀)	≥ 65	458	Serum	n/a	ng/mL	[162]
	Severe		n = 7, 4 (♂), 3 (♀)	≥ 65	956	-//-		-//-	
**Zn**									
	Control	Jigawa, Northwestern	n = 21	35.8	64.9	Plasma	n/a	µg/dL	[172]
	Positive	Nigeria	n = 50	43.8	58.1	-//-		-//-	
	Control	Chennai, India	n = 45	*32*	*105.8*	Serum	80-120	µg/dL	[38]
	Positive		n = 47	*34*	*74.5*	-//-		-//-	
	Control	Shoushtar city, Iran	n = 186	51	86.66	Serum	70-127	µg/dL	[173]
	Positive		n = 93		67.61	-//-		-//-	
	Control	Chennai, India	n = 45	*34 (18-77)*	*105.8*	Serum	n/a	µg/dL	[38]
	Positive		n = 47	*32 (18-60)*	*74.5*	-//-		-//-	
	Control	Moscow, Russia	n = 43, 27 (♂), 16 (♀)	55.67	0.96	Serum	n/a	µg/mL	[171]
	Mild		n = 50, 25 (♂), 25 (♀)	50.47	0.92	-//-		-//-	
	Moderate		n = 50, 31 (♂), 19 (♀)	54.22	0.90	-//-		-//-	
	Severe		n = 50, 25 (♂), 25 (♀)	64.5	0.87	-//-		-//-	
	Control	Germany	n = 35	*70*	975.7	Serum	n/a	µg/L	[80]
	Positive			*70*	717.4	-//-		-//-	
	Non-survivors			*89*	< 638.7	-//-		-//-	
	Non-severe	Riyadh, Saudi Arabia	n = 45	47.3	124.57	Serum	66-110	µg/dL	[157]

	Severe		n = 35	58.2	116.37	-//-		-//-	
	Non-severe	Wuhan, Hubei	n = 202, 88 (♂), 114 (♀)	*69*	6.61	WB	4.3-7.8	mg/L	[163]
	Severe	Province, China	n = 104, 60 (♂), 44 (♀)	*58*	6.18	-//-		-//-	
	Mild / Moderate	Sakai, Osaka, Japan	n = 22, 12 (♂)	≥ 65	87.7	Serum	n/a	μg/dL	[162]
	Severe		n = 7, 4 (♂)	≥ 65	62.4	-//-		-//-	
	Mild	Qena, Upper Egypt	n = 45, 24 (♂), 21 (♀)	31.8	0.67	Serum	n/a	μg/mL	[167]
	Common		n = 57, 33 (♂), 24 (♀)	47.8	0.62	-//-		-//-	
	Severe		n = 21, 15 (♂), 6 (♀)	59.1	0.73	-//-		-//-	
	Extremely severe		n = 11, 6 (♂), 5 (♀)	69.5	0.72	-//-		-//-	
	GCO group	n/a	n = 200	49.4	*970*	Plasma	n/a	μg/L	[158]
	PCO group		n = 75	64.1	*840*	-//-		-//-	
	Non-severe	Tabriz, Iran	n = 114, 58 (♂), 56 (♀)	56.72	68.42	Serum	72.6-127 (♂)	μg/dL	[174]
	Severe		n = 112, 56 (♂), 56 (♀)	56	67.30	-//-	77.0-114 (♀)	-//-	
	Recovered				69.66	-//-		-//-	
	Deceased				62.43	-//-		-//-	
	Non-severe	Wuhan, Hubei	n = 202, 88 (♂), 114 (♀)	*69*	*6.61*	WB	4.3-7.8	mg/L	[163]
	Severe	Province, China	n = 104, 60 (♂), 44 (♀)	*58*	*6.18*	-//-		-//-	
**Cu**									
	Control	Jigawa, Northwestern	n = 21	35.8	136.5	Plasma	n/a	μg/dL	[172]
	Positive	Nigeria	n = 50	43.8	128.3	-//-		-//-	
	Control	Moscow, Russia	n = 43, 27 (♂), 16 (♀)	55.67	1.13	Serum	n/a	μg/mL	[171]
	Mild		n = 50, 25 (♂), 25 (♀)	50.47	1.27	-//-		-//-	
	Moderate		n = 50, 31 (♂), 19 (♀)	54.22	1.46	-//-		-//-	
	Severe		n = 50, 25 (♂), 25 (♀)	64.5	1.33	-//-		-//-	
	Non-severe	Riyadh, Saudi Arabia	n = 45	47.3	18.35	Serum	10–22	μmol/L	[157]
	Severe		n = 35	58.2	18.2	-//-		-//-	
	Non-severe	Wuhan, Hubei	n = 202, 88 (♂), 114 (♀)	*69*	*838.55*	WB	634.1-999.4	μg /L	[163]
	Severe	Province, China	n = 104, 60 (♂), 44 (♀)	*58*	*929.73*	-//-		-//-	
	Survivors	Aschaffenburg-	n = 28, 13 (♂), 15 (♀)	*69*	1475.9	Serum	897.8-1906.0	μg /L	[175]
	Non-survivors	Alzenau, Germany	n = 7, 2 (♂), 5 (♀)	*89*	1317.9	-//-		-//-	
	Intermediate care group	n/a	n = 35	65	~ 140 $^{\#}$	Serum	n/a	μg/dL	[168]
	ICU group				< 100	-//-		-//-	
	Non-severe	Wuhan, Hubei	n = 70, 38 (♂), 32 (♀)	*60*	*15.84*	Urine	4-21.42	μg/L	[164]
		Province, China			*15.55*$^{\&}$	-//-	4.39-13.37$^{\&}$	μg/g	
	Severe		n = 68, 41(♂), 27 (♀)	*65*	*32.14*	-//-		μg/L	
					77.71$^{\&}$	-//-		μg/g	
**Se**									
	Control	India	n = 30, 14 (♂), 16 (♀)	*33.5 (37.5-43)*	79.1	Serum	70-150	ng/mL	[176]
	Positive		n = 30, 24 (♂), 6 (♀)	*40.5 (26-37)*	69.3	-//-		-//-	
	Control	Jigawa, Northwestern	n = 21	35.8	29.1	Plasma	n/a	ng/dL	[172]
	Positive	Nigeria	n = 50	43.8	25.3	-//-		-//-	
	Control	Moscow, Russia	n = 43, 27 (♂), 16 (♀)	55.67	0.102	Serum	n/a	μg/mL	[171]
	Mild		n = 50, 25 (♂), 25 (♀)	50.47	0.093	-//-		-//-	
	Moderate		n = 50, 31 (♂), 19 (♀)	54.22	0.090	-//-		-//-	

	Severe		n = 50, 25 (♂), 25 (♀)	64.5	0.087	-//-		-//-	
	Non-severe	Riyadh, Saudi Arabia	n = 45	47.3	134	Serum	70-150	µg/L	[157]
	Severe		n = 35	58.2	162	-//-		-//-	
	Non-severe	Wuhan, Hubei Province, China	n = 70, 38 (♂), 32 (♀)	*60*	*25.55*<br>*27.65*[&]	Urine<br>-//-	10.46-82.71<br>15.86-38.13[&]	µg/L<br>µg/g	[164]
	Severe		n = 68, 41 (♂), 27 (♀)	*65*	*20.27*<br>*45.63*[&]	-//-<br>-//-		µg/L<br>µg/g	
	Mild	Tehran, Iran	n = 23 (♂), 15 (♀)	51	47.07	Serum	70-150	ng/mL	[165]
	Moderate		n = 12 (♂), 15 (♀)	59	47.36	-//-		-//-	
	Severe		n = 12 (♂), 7 (♀)	81	29.86	-//-		-//-	
	Survivors	Alzenau, Germany	n = 27, 12 (♂), 15 (♀)	*69*	53.3	Serum	45.7–131.6	µg/L	[51]
	Non-survivors		n = 6, 2 (♂), 4 (♀)	*89*	40.8	-//-		-//-	
Se-P	Survivors	Alzenau, Germany	n = 27, 12 (♂), 15 (♀)	*69*	3.3	Serum	2.56–6.63	mg/L	[51]
	Non-survivors		n = 6, 2 (♂), 4 (♀)	*89*	2.1	-//-		-//-	
**Mn**									
	Control, Positive	Jigawa, Northwestern Nigeria	n = 21, n = 50	35.8, 43.8	2.10, 1.64	Plasma	n/a	mg/dL	[172]
	Non-severe	Wuhan, Hubei Province, China	n = 202, 88 (♂), 114 (♀)	*69*	12.96	WB	8.1-18.5	µg /L	[163]
	Severe		n = 104, 60 (♂), 44 (♀)	*58*	10.56	-//-		-//-	
	Non-severe	Wuhan, Hubei Province, China	n = 70, 38 (♂), 32 (♀)	*60*	*<0.53*<br>*0.32*[&]	Urine<br>-//-	<0.53-1.92<br>0.09-1.99[&]	µg/L<br>µg/g	[164]
	Severe		n = 68, 41(♂), 27 (♀)	*65*	*0.83*<br>*1.82*[&]	-//-<br>-//-		µg/L<br>µg/g	
**Cr**									
	Non-severe	Wuhan, Hubei Province, China	n = 202, 88 (♂), 114 (♀)	*69*	*0.81*	WB	<0.5-0.76	µg /L	[163]
	Severe		n = 104, 60 (♂), 44 (♀)	*58*	*1.03*	-//-		-//-	
	Non-severe	Wuhan, Hubei Province, China	n = 70, 38 (♂), 32 (♀)	*60*	*0.88*<br>*0.87*[&]	Urine<br>-//-	<0.24-0.5<br>0.05-0.43[&]	µg/L<br>µg/g	[164]
	Severe		n = 68, 41(♂), 27 (♀)	*65*	*1.96*<br>*4.01*[&]	-//-<br>-//-		µg/L<br>µg/g	
**Cd**									
	Non-severe	Wuhan, Hubei Province, China	n = 202, 88 (♂), 114 (♀)	*69*	*0.71*	WB	0.22 -6.44	µg /L	[163]
	Severe		n = 104, 60 (♂), 44 (♀)	*58*	*0.7*	-//-		-//-	
	Non-severe	Wuhan, Hubei Province, China	n = 70, 38 (♂), 32 (♀)	*60*	*0.95*<br>*1.01*[&]	Urine<br>-//-	0.34-3.39<br>0.27-2.23[&]	µg/L<br>µg/g	[164]
	Severe		n = 68, 41 (♂), 27 (♀)	*65*	*1.49*<br>*2.96*[&]	-//-<br>-//-		µg/L<br>µg/g	
**Hg**									
	Non-severe	Wuhan, Hubei Province, China	n = 202, 88 (♂), 114 (♀)	*69*	*1.68*	WB	<1.15-5.97	µg /L	[163]
	Severe		n = 104, 60 (♂), 44 (♀)	*58*	*1.45*	-//-		-//-	
	Non-severe	Wuhan, Hubei Province, China	n = 70, 38 (♂), 32 (♀)	*60*	*0.75*<br>*0.81*[&]	Urine<br>-//-	<0.72-2.16<br>0.15-1.62[&]	µg/L<br>µg/g	[164]
	Severe		n = 68, 41 (♂), 27 (♀)	*65*	*<0.72*<br>*1.54*[&]	-//-<br>-//-		µg/L<br>µg/g	
**Pb**									

	Non-severe	Wuhan, Hubei Province, China	n = 202, 88 (♂), 114 (♀)	*69*	*12.37*	WB	8.7-48.1	µg /L	[163]
	Severe		n = 104, 60 (♂), 44 (♀)	*58*	*10.57*	-//-		-//-	
	Non-severe	Wuhan, Hubei Province, China	n = 70, 38 (♂), 32 (♀)	*60*	*0.82*	Urine	0.24-2.29	µg/L	[164]
					0.86[&]	-//-	0.26-1.91[&]	µg/g	
	Severe		n = 68, 41 (♂), 27 (♀)	*65*	*0.83*	-//-		µg/L	
					1.85[&]	-//-		µg/g	

[*, **]All values are given as reported in the cited publications; [#] approximate value. n: number of patients; n/a: not available; Fe: iron; Zn: zinc; Cu: copper; Se: selenium; Mn: manganese; Cr: chromium; Cd: cadmium; Hg: mercury; Pb: lead; FER: ferritin; MeCon: metal concentration; WB: whole blood; Se-P: selenoprotein P; ICU: Intensive Care Unit; GCO group: good clinical outcome group; PCO group: poor clinical outcome group; [&] creatinine adjusted analyte level.

Table S2. Summary of results for macroelements: COVID-19 patients

Element	Patients (disease status)	Region	Number Sex	Age (mean/*median*/range)	MeCon (mean/*median*)[*]	Samples	Normal values[**]	Unit	Ref.
**Mg**									
	Non-severe	Wuhan, Hubei	n = 202, 88 (♂), 114 (♀)	*69*	*39.46*	WB	31.9- 48.0	mg/L	[163]
	Severe	province, China	n = 104, 60 (♂), 44 (♀)	*58*	*38.33*	-//-		-//-	
	Discharge	Tehran, Iran	n = 396, 271 (♂), 125 (♀)	60.4	1.83	Blood	1.5- 2.5	mmol/L	[187]
	Expire		n = 63, 49 (♂), 14 (♀)	70.6	1.61	-//-		-//-	
	Moderate	Nancy, France	n = 43, 16 (♂), 27 (♀)	67.4	0.73	Serum	0.85- 0.95	mmol/L	[186]
	Severe		n = 108, 63 (♂), 45 (♀)	71.6	0.77	-//-		-//-	
	Critical		n = 149, 104 (♂), 45 (♀)	63	0.79	-//-		-//-	
**Na**									
	Non-severe	Riyadh, Saudi	n = 45	47.3	138.0	Serum	n/a	mmol/L	[157]
	Severe	Arabia	n = 35	58.2	136.58	-//-		-//-	
	Mild	Zhejiang	n = 82	*49*	*139.6*	Serum	137- 147	mmol/L	[188]
	Severe	province, China	n = 9	*66*	*137.85*	-//-		-//-	
	Mild	Chongqing	n = 45	*44*	139	Plasma	n/a	mmol/L	[192]
	Severe	China	n = 40	*56*	136.5	-//-		-//-	
**K**									
	Control	Modena, Italy	n = 171	64.1	4.0	Serum	3.5-5.3	mmol/L	[198]
	Hypokalemic		n = 119	65.8	3.1	-//-		-//-	
	Non-severe	Riyadh, Saudi	n = 45	47.3	4.4	Serum	n/a	mmol/L	[157]
	Severe	Arabia	n = 35	58.2	4.2	-//-		-//-	
	Discharge	Tehran, Iran	n = 396, 271 (♂), 125 (♀)	60.4	3.95	Blood	3.6- 5	mmol/L	[187]
	Expire		n = 63, 49 (♂), 14 (♀)	70.6	3.65	-//-		-//-	
	Moderate / Mild	Wenzhou China	n = 135	*46*	3.5	Plasma	> 3.5	mmol/L	[155]
	Severe / Critical		n = 40		3.2	-//-		-//-	
	Mild	Chongqing	n = 45	*44*	4	Plasma	n/a	mmol/L	[192]
	Severe	China	n = 40	*56*	3.8	-//-		-//-	
**Ca**									
	Control	Hialeah, Florida	n = 72	36.9	< 10	Serum	8.8–10.2	mg/dL	[199]
	Non-severe		n = 72	37.5	< 9	-//-			
	Negative	Monza, Italy	n = 165	66 (18-101)	*2.28*	Serum	n/a	mmol/L	[154]
	Positive		n = 420		*2.15*	-//-		-//-	
	Negative	Shoushtar city,	n = 186	51	9.50	Serum	8.6- 10.3	mg/dL	[173]
	Positive	Iran	n = 93		9.14	-//-		-//-	

	Mild	Zhejiang	n = 82	*49*	*2.17*	Serum	2.11- 2.52	mmol/L	[188]
	Severe	province, China	n = 9	*66*	*2.01*	-//-		-//-	
	Non-severe	Wuhan, Hubei	n = 202, 88 (♂), 114 (♀)	*69*	*63.55*	WB	46.9- 66.8	mg/L	[163]
	Severe	province, China	n = 104, 60 (♂), 44 (♀)	*58*	*68.20*	-//-		-//-	
	Survivors	Blida, Algieria	n = 83	59.5	2.2	Serum	2.21-.2.48	mmol/L	[189]
	Non-survivors		n = 37	68.6	2.02	-//-		-//-	
**Ca^{2+}**									
	Negative	Monza, Italy	n = 165	66 (18-101)	*1.17*	WB	n/a	mmol/L	[154]
	Positive		n = 420		*1.12*	-//-		-//-	
**Cl**									
	Non-severe	Riyadh, Saudi	n = 45	47.3	103.3	Serum	n/a	mmol/L	[157]
	Severe	Arabia	n = 35	58.2	97.7	-//-		-//-	

[*, **]All values are given as reported in the cited publications. n: number of patients; n/a: not available; Mg: magnesium; Na: sodium; K: potassium; Ca: calcium; Cl: chloride; MeCon: metal concentration; WB: whole blood.

Table S3. Summary of clinical trials with zinc (Zn) for COVID-19

ClinTI	Official Title	Country	Agents	Zn compound	Zn dose	Frequency/Duration of Zn administration	Patient details
**Participants with COVID-19 / at high risk of COVID-19**							
NCT04370782*	A Randomized Study Evaluating the Safety and Efficacy of Hydroxychloroquine and Zinc in Combination With Either Azithromycin or Doxycycline for the Treatment of COVID-19 in the Outpatient Setting	USA	Zn/HCQ/AZM<br>Zn/HCQ/DOXY	ZnS	220 mg	OD for 5 d	n = 750<br>≥ 30 yr<br>♂/♀
NCT04342728*	Coronavirus Disease 2019- Using Ascorbic Acid and Zinc Supplementation (COVIDAtoZ) Research Study A Randomized, Open Label Single Center Study	USA	Zn and/or AscA<br>ST	ZnGLU	50 mg	OD at *h.s.* for 10 d	n = 520<br>≥ 18 yr<br>♂/♀
NCT04621461*	A Randomized, Placebo-Controlled Study Evaluating the Efficacy of Zinc for the Treatment of COVID-19 in the Outpatient Setting	USA	Zn<br>Placebo	ZnS	220 mg	OD for 5 d	n = 3<br>≥ 30 yr<br>♂/♀
CTRI/2020/05/025215*	A prospective, single centre, randomized open labelled comparative clinical study to evaluate the effectiveness of Siddha medicine, Kabasura kudineer and vitamin c-zinc supplementation in the management of asymptomatic COVID 19 patients.	India	KSK<br>VitC<br>Zn	n/a	100 mg	OD for 14 d	n = 60<br>18-55 yr<br>♂/♀
IRCT20180425039414N2*	The effect of zinc on the treatment and clinical course of patients with SARS-CoV-2 (COVID-19)	Iran	Zn<br>HCQ	n/a	220 mg	BID	n = 80<br>≥ 18 yr<br>♂/♀
NCT04446104*	A Randomized Open-label Prophylaxis Trial Among Migrant Workers at High-risk of COVID-19 (DORM Trial)	Singapure	Zn/VitC<br>HCQ<br>IVM<br>PI<br>VitC	n/a	80 mg	OD for 42d	n = 5000<br>21-60 yr<br>♂
NCT04584567*	COVID-19 Infection Prophylaxis With Low Dose of Doxycycline and Zinc in Health Care Workers	Tunisia	Zn + DOXY<br>DOXY (placebo)<br>Placebo	n/a	15 mg	daily	n = 1100<br>18-65 yr<br>♂/♀
NCT04468139†	The Study of Quadruple Therapy Zinc, Quercetin, Bromelain and Vitamin C on the Clinical Outcomes of Patients Infected With COVID-19	Saudi Arabia	Zn<br>QUE<br>BM<br>VitC	elemental	50 mg	daily	n = 60<br>≥ 18 yr<br>♂/♀
NCT04447534†	Does Zinc Supplementation Enhance the Clinical Efficacy of Chloroquine / Hydroxychloroquine in Treatment of COVID-19?	Egypt	HCQ + Zn<br>HCQ	n/a	n/a	n/a	n = 200<br>≥ 18 yr<br>♂/♀

NCT04384458[†]	Comparative Study of Hydroxychloroquine and Ivermectin in COVID-19 Prophylaxis	Brazil	HCQ+Zn<br>IVM+Zn	active Zn<br>active Zn	20 mg<br>20 mg	BID for 50 d<br>BID for 45 d	n = 400<br>18-70 yr<br>♂/♀
NCT04472585[†]	Sub-cutaneous Ivermectin in Combination With and Without Oral Zinc: a Placebo Randomized Control Trial on Mild to Moderate COVID-19 Patients	Pakistan	Zn/ST<br>IVM/ST<br>Zn + IVM/ST<br>Placebo/ST	ZnS	20 mg	TID	n = 180<br>18-60 yr<br>♂/♀
NCT04395768[†]	Therapies to Prevent Progression of COVID-19, Including Hydroxychloroquine, Azithromycin, Zinc, Vitamin D, Vitamin B12 With or Without Vitamin C, a Multi-centre, International, Randomized Trial: The International ALLIANCE Study	Australia	Zn<br>HCQ<br>AZM<br>VitD3<br>VitB12<br>(+)/(-) VitC	ZnCIT	30 mg	PO daily	n = 200<br>≥ 18 yr<br>♂/♀
NCT04334512[†]	A Randomized, Double-Blind, Placebo-Controlled Phase IIa Study of Quintuple Therapy to Treat COVID-19 Infection	USA	Zn<br>HCQ<br>AZM<br>VitC<br>VitD<br>Zn, VitC, D (placebo)	n/a	n/a	10 d	n = 600<br>≥ 18 yr<br>♂/♀
NCT04621149[†]	A Phase 2 Screening Study of Candidate Non-prescription Treatments for COVID-19: A Patient-driven, Randomized, Factorial Study Evaluating Patient-reported Outcomes (PROFACT-01)	USA	Zn<br>ClO_2<br>FMT<br>LF<br>GTE<br>Placebo	ZnACE	n/a	7 d	n = 120<br>20-70 yr<br>♂/♀
NCT04641195[†]	A Randomized Trial to Determine the Effect of Vitamin D and Zinc Supplementation for Improving Treatment Outcomes Among COVID-19 Patients in India	India	Zn + VitD3<br>Zn (placebo)<br>VitD3 (placebo)<br>Placebo	ZnGLU	40 mg	OD up to 8 wk	n = 700<br>≥ 18 yr<br>♂/♀
NCT04323228[†]	Anti-inflammatory/Antioxidant Oral Nutrition Supplementation on the Cytokine Storm and Progression of COVID-19: A Randomized Controlled Trial	Saudi Arabia	ONS with Zn<br>Placebo	n/a	5.7 mg	14 d	n = 40<br>18-65yr<br>♂/♀
NCT04335084[†]	A Randomized, Double-Blind, Placebo-Controlled Phase IIa Study of Hydroxychloroquine, Vitamin C, Vitamin D, and Zinc for the Prevention of COVID-19 Infection	USA	Zn, HCQ,VitC,VitD<br>Zn, VitC, D (placebo)	n/a	n/a	12 wk	n = 600<br>≥ 18 yr<br>♂/♀
ACTRN126200000454976[†]	High-dose intravenous zinc (HDIVZn) as adjunctive therapy in COVID-19 positive critically ill patients: A pilot randomized controlled trial	Australia	Zn<br>Placebo	ZnC	0.5 mg/kg	daily for 7 d	n = 160<br>≥ 18 yr<br>♂/♀
NCT04558424[#]	Randomized, Double -Blind, Placebo Controlled, Trial to Evaluate the Effect of	Bangladesh	Zn + AscA/ST<br>Placebo/ST	ZnGLU	220 mg	OD for 10 d	n = 50<br>18-70 yr

	Zinc and Ascorbic Acid Supplementation in COVID-19 Positive Hospitalized Patients in BSMMU						♂/♀
PACTR202005622389003[#]	Efficacy and safety of Hydroxychloroquine, Azythromycine and Zinc for the treatment of patients with SARS-Cov2 infection in Senegal: a dose ranging randomised trial. COVID-19	Senegal	Zn/ST<br>HCQ+AZM	n/a	20 mg	OD for 6 d	n = 128<br>18-65 yr<br>♂/♀
CTRI/2020/07/026340[#]	Prospective study to assess therapeutic role of Zinc in COVID-19 patients	India	Zn/ST	ZnS	100 mg	OD	n = 100<br>18-80 yr<br>♂/♀
NCT04898023[#]	Evaluation of Combination Zinc and Green Tea Extract Supplementation on Reduction in Symptom Duration and Severity Associated With Community Respiratory Viral Infections: a Randomized Control Trial (ZiPhenol Study)	USA	Zn+GTE+AscA<br>Placebo	ZnCIT	50 mg	2 cap BID for 5 d	n = 100<br>≥ 18 yr<br>♂/♀
NCT04377646[#]	A Study of Hydroxychloroquine and Zinc in the Prevention of COVID-19 Infection in Military Healthcare Workers (COVID-Milit)	Tunisia	Zn<br>Zn (placebo)<br>HCQ<br>HCQ (placebo)	elemental	15 mg	OD up to 2 mo	n = 660<br>18-65 yr<br>♂/♀
NCT04542993[††]	Can SARS-CoV-2 Viral Shedding in COVID-19 Disease be Reduced by Resveratrol-assisted Zinc Ingestion, a Direct Inhibitor of SARS-CoV-2-RNA Polymerase? A Single Blinded Phase II Protocol (Reszinate Trial)	USA	Zn + RSV<br>Zn + RSV (placebo)	ZnPIC	50 mg	TID for 5 d	n = 60<br>18-75 yr<br>♂/♀
NCT04482686[††]	A Phase I Double-Blind Randomized Placebo-Controlled Trial of Combination Therapy to Treat COVID-19 Infection	USA	Zn<br>IVM<br>DOXY-Hcl<br>VitC<br>VitD3<br>Zn, VitC, D3 (placebo)	ZnS	n/a	10 d	n = 31[&]<br>≥ 18 yr<br>♂/♀

ClinTI: Clinical Trial Identifier; n: number of patients; yr: year; mo: month; d: day; cap: capsules; *h.s.*: *hora somni*; OD: once daily; BID: twice a day; TID: three times a day; PO: *per os*; BSMMU: Bangabandhu Sheikh Mujib Medical University; QUE: quercetin; BM: bromelain; HCQ: hydroxychloroquine; IVM: ivermectin; DOXY: doxycyclin; DOXY-Hcl: doxycycline hyclate; FMT: famotidine; LF: lactoferrin; RSV: resveratrol; AZM: azithromycin; AscA: ascorbic acid; ClO_2: chlorine dioxide; PI: povidone iodine; KSK: Kabasura Kudineer; VitB12: vitamin B12; VitC: vitamin C; VitD: vitamin D; VitD3: vitamin D3; Zn: zinc; GTE: green tea extract; ZnGLU: zinc gluconate; ZnCIT: zinc citrate; ZnPIC: zinc picolinate; ZnS: zinc sulfate; ZnC: zinc chloride; ZnACE: zinc acetate; ONS: oral nutrition suplement; (+): with, (-): without ST: standard treatment; n/a: not available. Recruitment status: * complete, [†] recruiting, [#] not yet recruiting, [††] active, not recruiting . [&] Actual enrollment.

Table S4. Summary of clinical trials with copper (Cu) for COVID-19

ClinTI	Official Title	Country	Agents	Cu dose	Frequency/Duration of Cu administration	Patient details
**Participants with COVID-19**						
CTRI/2020/07/026514#	A Phase-II, Open Label, Randomized Controlled Trial of Resveretrol-Copper Plus Standard Treatment Or Sodium-Copper-Chlorophyllin Plus Standard Treatment Versus Standard Treatment in Cancer patients with SARS-CoV-2 Infection who are Symptomatic Or have Pneumonia for COVID 19	India	RSV-Cu/ST<br>CHLN/ST<br>ST without RSV-Cu	560 ng	1 tablet once every 6 hr from the date of randomization	n = 200<br>18-99 yr<br>♂/♀
CTRI/2020/05/025337#	A Phase-II, Open Label, Randomized Controlled Trial Of Resveretrol-Copper Plus Standard Treatment Or Sodium-Copper-Chlorophyllin Plus Standard Treatment Versus Standard Treatment In Hospitalized Patients With Pneumonia Due To SARS-CoV-2 (COVID-19)	India	RSV-Cu/ST<br>CHLN/ST<br>ST without RSV-Cu	560 ng	1 tablet QID from the date of randomization	n = 200<br>18-99 yr<br>♂/♀
CTRI/2020/05/025336#	A Phase-III, Open Label, Randomized Controlled Trial Of Resveretrol-Copper Plus Standard Treatment Or Sodium-Copper-Chlorophyllin Plus Standard Treatment Versus Standard Treatment In Asymptomatic Or Mildly Symptomatic Patients With SARS-CoV-2 Infection (COVID-19)	India	RSV-Cu/ST<br>CHLN/ST<br>ST without RSV-Cu	560 ng	1 tablet QID from the date of randomization	n = 300<br>18-99 yr<br>♂/♀

ClinTI: Clinical Trial Identifier; n: number of patients; hr: hours; yr: year; ST: standard treatment; RSV-Cu: resveratrol-copper tablets; CHLN: chlorophyllin; QID: four times a day.
Recruitment status: # not yet recruiting.

Table S5. Summary of clinical trials with selenium (Se) for COVID-19

ClinTI	Official Title	Country	Agents	Se dose	Frequency/Duration of Se administration	Patient details
**Participants with COVID-19**						
IRCT20210427051100N1*	The effect of selenium supplementation on inflammatory markers and blood cells in patients with COVID-19 double-blind randomized clinical trial	Iran	Se/ST<br>CG/Placebo/ST	200 µg	daily for 14 d	n = 40<br>20-60 yr<br>♂/♀
IRCT20190418043307N1*	Investigating the effectiveness of selenium on recovery of hospitalized patients with COVID-19	Iran	Se/ST<br>CG/ST	200 µg	daily for 14 d	n = 100<br>18-65 yr<br>♂/♀
IRCT20160706028815N5*	The effect of addition of selenium to treatment regimen of hospitalized patients with COVID-19 on the outcome of disease compared with control group	Iran	Se/ST<br>CG/ST	1000 µg / 500 µg	every 12 hr on the 1st d / every 12 d from the 2nd d	n = 40<br>≥ 18 yr<br>♂/♀
IRCT20190312043030N2*	The effect of combination of selenium, vitamin C and methylprednisolone in acute respiratory distress syndrome mortality and morbidity from COVID-19	Iran	Se/VitC/MPS/ST<br>CG/ST	1 mg	daily for 7 d	n = 40<br>18-90 yr<br>♂/♀
IRCT20160919029870N3*	Evaluation of the effectiveness of selenium added to intravenous nutrition therapy on mortality and duration of ICU hospitalization in patients with COVID-19 disease	Iran	Se<br>CG/ST	1 mg / 500 µg	at the beginning / IVI daily during 15 min at 2 PM every d for 5 d	n = 80<br>no age limit<br>♂/♀
NCT04869579#	Selenium as a Potential Treatment for Moderately-ill, Severely-ill, and Critically-ill COVID-19 Patients	USA	SeA/ST<br>Placebo/ST	2000 µg<br>1000 µg	on d 1<br>d 2-14	n = 100<br>≥ 18 yr<br>♂/♀

ClinTI: Clinical Trial Identifier; n: number of patients; d: day; hr: hours; min: minutes; Se: selenium; ST: standard treatment; CG: control group; VitC: vitamin C; MPS: methylprednisolone; IVI: intravenous infusion. Recruitment status: * complete, # not yet recruiting.

Table S6. Summary of clinical trials with magnesium (Mg) for COVID-19

ClinTI	Official Title	Country	Agents	Mg compound	Mg dose	Frequency/Duration of Mg administration	Patient details
**Participants with COVID-19**							
IRCT20191211045691N1*	Evaluation of the efficacy and safety of inhaled magnesium sulfate in combination with standard treatment in COVID-19 patients: a clinical trial	Iran	Mg/ST<br>CG/ST	MgS	5 cc of a 20% INJ-V or 2 cc of a 50% INJ-V	every 8 h for 5 d	n = 100<br>18-80 yr<br>♂/♀
IRCT20210702051763N1*	The effect of vitamin D and magnesium supplementation on clinical symptoms, inflammatory markers and oxidative stress in patients with COVID-19: double-blind randomized control clinical trial	Iran	Mg+VitD<br>VitD+Mg (placebo)<br>Mg+VitD (placebo)<br>Placebo	n/a	300 mg	for 3 wk	n = 104<br>18-65 yr<br>♂/♀
NCT04941703[†]	Investigation of Choice Alteration of the Gut Metagenome on COVID-19 Severity	USA	Mg/PB<br>Placebo	MgCIT	296 ml	1 bottle PO once within a 4-h period	n = 30<br>18-99 yr<br>♂/♀
CTRI/2020/06/026189[#]	Randomized, Double Blind, Parallel Group Study of Vitamin D3 & Magnesium in COVID-19 Infection	India	VitD3+Mg	MgGly	250 mg	BID for 14 d	n = 210<br>20-60 yr<br>♂/♀

ClinTI: Clinical Trial Identifier; n: number of patients; wk: weeks; d: day; h: hour; BID: twice a day; PO: *per os*; PB: probiotic; INJ-V: injectable vial; cc: cubic centimeter; MgS: magnesium sulfate; MgCIT: magnesium citrate; MgC: magnesium carbonate; MgGly: magnesium glycinate VitD: vitamin D; ST: standard treatment; CG: control group. Recruitment status: * complete, [†] recruiting, [#] not yet recruiting.
